# Novel Primary Human Cancer Stem-Like Cell Populations from Non-Small Cell Lung Cancer: Inhibition of Cell Survival by Targeting NF-κB and MYC Signaling

**DOI:** 10.3390/cells10051024

**Published:** 2021-04-27

**Authors:** Beatrice A. Windmöller, Morris Beshay, Laureen P. Helweg, Clara Flottmann, Miriam Beermann, Christine Förster, Ludwig Wilkens, Johannes F. W. Greiner, Christian Kaltschmidt, Barbara Kaltschmidt

**Affiliations:** 1 Department of Cell Biology, University of Bielefeld, Universitätsstrasse 25, 33615 Bielefeld, Germany; L.Helweg@uni-bielefeld.de (L.P.H.); Clara.Raeker@uni-bielefeld.de (C.F.); miriam.beermann@student.uni-luebeck.de (M.B.); Johannes.greiner@uni-bielefeld.de (J.F.W.G.); C.Kaltschmidt@uni-bielefeld.de (C.K.); barbara.kaltschmidt@uni-bielefeld.de (B.K.); 2 Forschungsverbund BioMedizin Bielefeld/OWL FBMB e. V., Maraweg 21, 33617 Bielefeld, Germany; morris.beshay@evkb.de (M.B.); christine.foerster@krh.eu (C.F.); ludwig.wilkens@krh.eu (L.W.); 3 Department of General Thoracic Surgery, Protestant Hospital of Bethel Foundation, Burgsteig 13, 33617 Bielefeld, Germany; 4 Institute of Pathology, KRH Hospital Nordstadt, Haltenhoffstrasse 41, Affiliated with the Protestant Hospital of Bethel Foundation, 30167 Hannover, Germany; 5 Molecular Neurobiology, Bielefeld University, Universitätsstrasse 25, 33615 Bielefeld, Germany

**Keywords:** cancer stem cell-like cells, squamous cell carcinoma, adenocarcinoma, NSCLC, MYC, NF-κB

## Abstract

There is growing evidence that cancer stem cells (CSCs), a small subpopulation of self-renewal cancer cells, are responsible for tumor growth, treatment resistance, and cancer relapse and are thus of enormous clinical interest. Here, we aimed to isolate new CSC-like cells derived from human primary non-small cell lung cancer (NSCLC) specimens and to analyze the influence of different inhibitors of NF-κB and MYC signaling on cell survival. CSC-like cells were established from three squamous cell carcinomas (SCC) and three adenocarcinomas (AC) of the lung and were shown to express common CSC markers such as Prominin-1, CD44-antigen, and Nestin. Further, cells gave rise to spherical cancer organoids. Inhibition of MYC and NF-κB signaling using KJ-Pyr-9, dexamethasone, and pyrrolidinedithiocarbamate resulted in significant reductions in cell survival for SCC- and AC-derived cells. However, inhibition of the protein–protein interaction of MYC/NMYC proto-oncogenes with Myc-associated factor X (MAX) using KJ-Pyr-9 revealed the most promising survival-decreasing effects. Next to the establishment of six novel in vitro models for studying NSCLC-derived CSC-like populations, the presented investigations might provide new insights into potential novel therapies targeting NF-κB/MYC to improve clinical outcomes in NSCLC patients. Nevertheless, the full picture of downstream signaling still remains elusive.

## 1. Introduction

Lung cancer is the leading cause of cancer death worldwide and can be broadly classified into two types: small cell lung cancers (SCLC) and non-small cell lung cancers (NSCLC) [1,2]. The most common type of lung cancer is NSCLC, which accounts for approximately 80–85% of all lung cancer cases and can be divided into adenocarcinoma (AC, 40%), squamous cell carcinoma (SCC, 25–30%), and large cell carcinoma (5–10%) [3,4]. Even if NSCLC is less aggressive than SCLC and death rates for lung cancer in general dropped by 48% from 1990 to 2016 among males and by 23% from 2002 to 2016 among females, NSCLC prognosis is still poor, with an age-standardized 5-year net survival of approximately 19% [1]. This poor prognosis is mainly caused by cancer aggressiveness and therapy resistance, which is increased by genetic and phenotypic heterogeneity of lung cancer cells [5].

There is growing evidence that a small subpopulation of cancer cells with stem cell characteristics, so-called cancer stem cells (CSCs), are responsible for tumor growth, therapy resistance, and recurrence, as well as metastasis, probably by remodeling the process of epithelial–mesenchymal transition (EMT) in lung cancer [6] (reviewed in [7,8]). Thus, CSCs are of enormous clinical interest and human in vitro models are extremely important to gather more insights into molecular regulators of lung cancer. Next to the formation of spherical cancer organoids [9], lung cancer stem cells (LCSCs) can be identified using different markers such as cell surface glycoproteins Prominin-1 (CD133) and CD44-antigen (CD44) [10,11,12]. These CSCs markers are linked to increased chemoresistance and poor prognosis as well as reduced overall survival in patients with lung cancer [12,13,14]. Other important regulators of CSC characteristics are members of the MYC transcription factor family, consisting of L-, N-, and C-MYC (further referred as MYC within this manuscript). Within a recent pan-cancer study, Schaub and coworkers identified MYC family members amplified in up to 33% of all lung adenocarcinomas [15]. Furthermore, MYC amplification was described as a poor prognostic marker of early stage adenocarcinomas of the lung [16] as well as MYC gain determined by fluorescence in situ hybridization was shown to be an independent poor prognostic factor for disease-free survival and overall survival in lung adenocarcinomas [17]. Concerning LCSCs, suppression of Myc proto-oncogene (MYC) signaling was shown to reduce viability, self-renewal, and invasion capacity of LCSC-like cells derived from A549 cells [18]. Accordingly, the LCSC phenotype of H460 and H292 cells was shown to be impaired by the suppression of MYC via the Src-STAT3 pathway [19]. Despite these promising findings, the role of MYC signaling in primary human LCSCs still remains unknown.

Next to the re-regulation of diverse stemness associated genes, transcriptomic profiling revealed a significant involvement of the transcription factor nuclear factor kappa-light-chain-enhancer of B cells (NF-κB) in regulating LCSC populations [10]. NF-κB signaling is vital for a broad range of cellular processes including proliferation, differentiation, apoptosis, immune response, angiogenesis, and inflammation [20,21]. Moreover, NF-κB activation is associated with cancer development, pathogenesis, abnormal cell proliferation and differentiation, enhanced metastasis, and treatment resistance in several tumors [22,23,24,25,26]. A meta-analysis further indicated that higher NF-κB expression in NSCLC cells is associated with shorter overall survival of NSCLC patients and is closely correlated with tumor stage, lymph node metastasis, and 5-year overall survival [27].

In the present study, we established three primary SCC-derived lung cancer cell populations named BKZ-4, BKZ-5, and BKZ-6, as well as three primary AC-derived lung cancer cell populations named BKZ-7, BKZ-8, and BKZ-9. All isolated NSCLC cell populations were enriched for a subset of cells with markers of stemness and were able to form spherical cancer organoids. Application of the NF-κB inhibitors dexamethasone and pyrrolidinedithiocarbamate (PDTC) significantly decreased cell survival of AC- and SCC-derived cells, while the NF-κB inhibitor lenalidomide did not impair cell survival. Notably, exposure of AC- and SCC-derived CSCs to the small molecule KJ-Pyr-9, which inhibits the protein–protein interaction of MYC/N-myc proto-oncogene (NMYC) with Myc-associated factor X (MAX) [28], resulted in the strongest decrease in cell survival.

## 2. Materials and Methods

### 2.1. Lung Cancer Stem Cell-like Cell Population Establishment and Cell Culture

The cancer tissue samples used to isolate lung cancer stem cell-like cells were obtained during surgical resection and were kindly provided by the Forschungsverbund BioMedizin Bielefeld/OWL FBMB e. V. (Bielefeld, Germany) at the Protestant Hospital of Bethel Foundation (Bielefeld, Germany) after assuring routine histopathological analysis. Informed consent according to local and international guidelines was signed by all patients. All further experimental procedures were ethically approved (Ethics Committee Münster, Germany, 2017-522-f-S).

To obtain tumor material for the isolation of primary cells, we collected a cubic sample from each tumor type and transferred to ice-cold Dulbecco’s phosphate-buffered saline (Sigma Aldrich, München, Germany). The specimen was washed 10 times with ice-cold PBS, mechanically disintegrated into 1–2 mm pieces, and enzymatically digested with Collagenase for 2 h at 37 °C as previously described [29]. The minced tissue was cultivated in cancer stem cell medium comprising Dulbecco’s modified Eagle’s medium/Ham’s F-12 (Sigma Aldrich, München, Germany) with the addition of 2 mM L-glutamine (Sigma Aldrich), penicillin/streptomycin (100 μg/mL; Sigma Aldrich, München, Germany), epidermal growth factor (EGF) (20 ng/mL; Miltenyi Biotec, Bergisch Gladbach, Germany), basic fibroblast growth factor (FGF-2) (40 ng/mL; Miltenyi Biotec, Bergisch Gladbach), B27 supplement (Gibco, Thermo Fisher Scientific, Bremen, Germany), and 10% fetal calf serum (FCS) (Sigma Aldrich, München, Germany) in T75 culture flasks coated with 0.1% gelatin from bovine skin (type B; Sigma Aldrich, München, Germany). Adult human dermal fibroblasts (HDFs) (Genlantis, San Diego, CA, USA) and the well-established lung adenocarcinoma-derived cell line LXF-289 [30] (DSMZ, Braunschweig, Germany) were cultured in Dulbecco’s modified Eagle’s medium-high glucose (Sigma Aldrich, München, Germany) with 10% FCS, 2 mM L-glutamine, and penicillin/streptomycin (100 µg/mL). All cells were cultured at 37 °C and 5% CO_2_ in a humidified incubator. Adherent cells were harvested with 0.05% Trypsin–EDTA solution (0.5 mg/mL; Sigma Aldrich, München, Germany). BKZ populations and LXF-289 cells were kept in culture for 1 week before experiments were conducted as well as only confluent cells were passaged. For sphere formation, 0.5 × 10^6^ cells were cultured in CSC medium supplemented with 4 µg/mL heparin (Sigma Aldrich, München, Germany) in low adhesion T25 culture flask.

Population doubling times were determined using the Orangu Cell Counting Solution (Cell Guidance Systems, Cambridge, UK) and were performed nine times per cell population according to the manufacturers guidelines. For the standard curve, 1000, 2500, 5000, 7500, and 10,000 cells per 100 µL medium supplemented with 10% FCS were seeded in a 0.1% gelatin coated 96 well-plate. To determine the doubling time, we seeded 3000 cells per 100 µL medium supplemented with 10% FCS in a 0.1% gelatin-coated 96-well plate and cultivated for 72 h. Cell viability was measured and cell count was quantified using the respective standard curve. Growth rate and populations doubling times were determined by the following equations:

(1) $$growth\;rate=\frac{\ln\left(xt\right)\;-\;\ln\left(x0\right)}{t\;-\;t0}$$

(2) $$population\;doubling\;time=\frac{\ln\left(2\right)}{growth\;rate}$$

### 2.2. Immunocytochemistry

For immunocytochemical staining, pre-cultured BZK-4, BZK-5, BZK-6, BZK-7, BZK-8, BZK-9, LXF-289 cells, and HDFs were harvested and 1.5 × 10^4^ cells per 500 µL medium supplemented with 10% FCS were seeded on etched cover slips in a 24-well plate. When 80% confluency was reached, cells were fixed with 4% phosphate-buffered paraformaldehyde (lab-made) for 15 min at room temperature (RT). After three washing steps with 1 × PBS, cells were blocked and permeabilized using 0.02% Triton-X 100 (Sigma Aldrich) with 5% goat serum (Dianova, Hamburg, Germany) for 30 min at RT followed by incubation with the primary antibody for 1 h at RT. Antibodies used were anti-CD44 (1:400; 156-3C11; Cell Signaling, Frankfurt am Main, Germany), anti-CD133 (1:100; NB120-16518; NovusBio, Bio-Techne, Wiesbaden-Nordenstadt, Germany), anti-Nestin (1:200; MAB5326; Millipore, Merck, Darmstadt, Germany), anti-MYC (0.1 μg/mL; Y69; Abcam), anti-NMYC (2.5 μg/mL; NCM II 100; Abcam), anti-RelA (1:400; Cell Signaling), anti-RelB (1:100; Cell Signaling), and anti-cRel (1:100; Cell Signaling). Afterwards, secondary fluorochrome-conjugated antibodies (1:300; goat anti-mouse Alexa 555, goat anti-rabbit Alexa 555, goat anti-mouse Alexa 488; Life Technologies, Thermo Fisher Scientific) were incubated for 1 h at RT in the dark, followed by nuclear counterstaining with 4′,6-diamidino-2-phenylindole (DAPI; 1 μg/mL; Sigma Aldrich) for 10 min at RT. Fluorescence imaging was conducted using a confocal laser scanning microscope (LSM 780; Carl Zeiss, Jena, Germany) and analyzed using ZEN software from the same provider or Fiji ImageJ [31]. For the quantification of the amount of CD133/CD44 positive cells, the percentage of CD133/CD44 double-positive cells was conducted for at least five images per cell population.

For immunocytochemical staining of spheres, spheres were harvested at 300× *g* for 10 min, fixated in 4% PFA for two hours, and washed with water for 15 min. Afterwards, spheres were incubated in 50% isopropanol for 45 min, 75% isopropanol for 1 h, 90% isopropanol for 1 h, and finally 100% for 1 h and 15 min. In the final step, 100% isopropanol was changed after 1 h. Between each step, the spheres were harvested at 300× *g* for 10 min. After isopropanol vaporized, spheres were overnight embedded in paraffin (Sigma Aldrich) and centrifuged at 450× *g* for 10 min. Paraffin-embedded sections were washed twice in xylol for 10 min followed by 100% ethanol for 10 min. Thereafter, sections were rehydrated by 5 min washing steps in 90% ethanol, followed by 80% ethanol and 70% ethanol. Epitope retrieval was performed by boiling the slides in 0.01 M citrate buffer, pH 6.0 (lab made), for 20 min. After cooling down for at least 30 min at RT, the slides were washed twice with 0.02% Triton-X 100. Afterwards, slides were blocked and permeabilized using 0.02% Triton-X 100 with 10% goat serum and 1% bovine serum albumin for 2 h at RT. Anti-CD133 (1:100; NB120-16518; NovusBio), anti-CD44 (1:400; 156-3C11; Cell Signaling), anti-MYC (5 µg/mL; Y69, Abcam), and anti-NMYC (5 µg/mL; NCM II 100; Abcam) first antibodies were diluted in blocking solution and incubated over night at 4 °C. After three washing steps with PBS, secondary fluorochrome-conjugated antibodies (1:300; goat anti-mouse Alexa 555, goat anti-rabbit Alexa 555; Life Technologies) were applied and incubated for 1 h at RT in the dark. After three washing steps with PBS, nuclear counterstaining was performed using DAPI (1 μg/mL) for 10 min at RT. Fluorescence imaging was performed using a confocal laser scanning microscope (LSM 780; Carl Zeiss) and analyzed using Fiji ImageJ.

To determine the nuclear size of the cells, we analyzed five randomized images of immunocytochemically stained cells for each cell population. The area of each nucleus was defined in the DAPI channel and was measured using ImageJ. The size of the nuclei were clustered into three groups: (1) ≤100 µm^2^, (2) ≥100 to ≤200 µm^2^, and (3) ≥200 µm^2^.

For the tumor necrosis factor α (TNF-α) treatment, 2 × 10^4^ cells were seeded in 500 µL CSC medium supplemented with 10% FCS on etched cover slips. After 1 day of cultivation, medium was replaced with CSC medium supplemented with 10% FCS and 10 ng/mL TNFα (Miltenyi Biotec). Subsequent to the incubation for 10, 30, and 60 min, cells were each washed with 1 × PBS and fixated with 4% PFA for 15 min. As control, cells were incubated in CSC medium supplemented with 10% FCS without TNF-α for 60 min. After fixation, cells were washed with 1 × PBS and immunocytochemically stained for RelA as described above. To quantify the fluorescence intensity (FI) in the nuclei, we took five randomized pictures for each time point and cell population. The area of each nucleus was defined in the DAPI channel using ImageJ and the average nuclear fluorescence intensity of the respective protein channel was measured by overlay. The fluorescence intensities of all nuclei with an area value of ≥30 µm^2^ were included in the quantification. The fold change of the nuclear fluorescence intensity was calculated according to the following equation:

(3) $$\mathrm{fold}\;\mathrm{change}\;=\frac{FI_{ex}-FI_{min}}{FI_{max}-FI_{min}}\;*\;100$$

The ratio of nuclear to total fluorescence intensity (N/T ratio) was calculated according to Kelley and Paschal [32]. To measure the total fluorescence intensity, we placed a ring measuring 1,098,159 µm^2^ around the nucleus of the cell, and the average fluorescence intensity was determined. The average nuclear fluorescence intensity was measured as described above, and the ratio of nuclear to total fluorescence intensity was calculated accordingly.

For the immunocytochemical staining of MYC after KJ-Pyr-9-treatment, 1.5 × 10^4^ cells per 500 µL CSC medium containing 10% FCS were seeded in a 24 well on top of etched cover slips. After adherence (4–6 h), cells were treated with 10 µM and 20 µM of KJ-Pyr-9 or dimethyl sulfoxide (DMSO) as a control for 24 h. Afterwards, immunocytochemical staining against MYC was performed and nuclear fluorescence intensity was measured as described above. For the immunocytochemical staining of RelA after PDTC-treatment, 1.5 × 10^4^ cells per 500 µL CSC medium containing 10% FCS were seeded in a 24 well on top of etched cover slips. After adherence (4–6 h), cells were treated with/without 100 µM PDTC for 24 h. Thereafter, immunocytochemical staining against RelA was conducted and ratio of nuclear to total fluorescence intensity was measured as described above.

### 2.3. Western Blot

For preparation of whole cell lysates, 10^6^ cells were lysed in 0.1 M Tris, 3 mM EDTA, and 1% SDS. For each cell population, 20 µg protein was used and separated by SDS-PAGE followed by the transfer to a PVDF membrane (Carl Roth GmbH, Karlsruhe, Germany). After the membrane was washed with 0.05% Tween-20 (VWR International GmbH, Darmstadt, Germany) in PBS (lab-made) for 10 min three times, the membrane was blocked using PBS containing 0.05% Tween-20 and 5% milk powder (Carl Roth GmbH) followed by probing with primary antibodies anti-MYC (1:1000, Y69, Abcam), anti-NMYC (1:50, NCM II 100; Abcam), and anti β-Actin (1:1000, 13E5, Cell Signaling) overnight at 4 °C. After three washing steps, the secondary horseradish peroxidase-conjugated antibody (anti-rabbit or anti-mouse, 1:4000, DIANOVA) was applied for 1 h at RT. Then, the membrane was washed again three times using PBS containing 0.05% Tween-20 with an additional washing step in 10 mM Tris-HCl (pH 7.5). Subsequent to the development, we used enhanced chemiluminescence with a solution containing 1 mL of Solution A (50 mg Luminol; Sigma-Aldrich in 200 mL 0.1 M Tris-HCl), 0.3 µL 30% H_2_O_2_, and 100 µL Solution B (11 mg Coumarin acid; Sigma-Aldrich in 10 mL DMSO) on a radiographic film (Super RX-N, FUJIFILM, Düsseldorf, Germany). The protein amounts were normalized to their related β-actin signals and quantified using ImageJ and Prism V5.01 software.

### 2.4. Senescence Assay

To measure the number of senescent cells, we seeded 5 × 10^4^ cells per 2 mL CSC medium supplemented with 10% FCS in a 0.1% gelatin-coated 6 well-plate. After adherence (4–6 h), cells were each treated with 20 µM KJ-Pyr-9, 100 µM PDTC, and DMSO as a control for 24 h. Then, activity of senescence-associated β-galactosidase was measured according to Debacq-Chainiaux and colleagues [33]. Briefly, cells were washed with PBS and fixated with 3% PFA; then, the staining solution containing 1 mg/mL X-Gal (Carl Roth GmbH) was added. Incubation overnight at 37 °C led to final staining, which could be visualized by phase contrast microscopy. For each condition and each cell line, three pictures were taken and analyzed, and percentage of senescent cells was calculated accordingly.

### 2.5. Quantitaive Polymerase Chain Reaction

For the analysis of gene copy number of NMYC and MYC, we isolated genomic DNA using the QUIamp DNA Mini Kit (Qiagen, Hilden, Germany) according to the manufacturer’s guidelines. Gene copy number was quantified using the Platinum SYBR Green qPCR Super-Mix UDG (Invitrogen, Thermo Fisher Scientific) according to the manufacturer’s guidelines. Each copy number quantification was performed in triplicate and was assayed with a Rotor Gene 600 (Qiagen). The gene assays each included a no-template control, 10 ng of calibrator human genomic DNA (Sigma Aldrich), and 10 ng of CSC DNA. Haploid copy number was determined according to De Preter et al. [34].

For the analysis of MYC target genes under the influence of KJ-Pyr-9, we seeded 7.5 × 10^4^ BKZ-6, BKZ-8, and LXF-289 cells in a 6-well plate. After 1 day of cultivation, cells were treated with 20 µM KJ-Pyr-9 for 24 h. Thereafter, cells were harvested using a cell scrapper, and RNA was isolated using the NucleoSpin RNA Kit (Macherey-Nagel, Düren, Germany) according to the manufacturer’s guidelines. Quality and concentration of RNA were assessed via Nanodrop ultraviolet spectrophotometry. Copy DNA (cDNA) synthesis was performed using 250 ng of RNA and the First Strand cDNA Synthesis Kit (Thermo Fisher Scientific). For the synthesis, random hexamer primers were used. Quantitative polymerase chain reaction was performed in triplicates using the qPCRBIO Sygreen-Mix (PCR Biosystems, London, UK) according to the manufacturer’s guidelines and assayed with a Rotor Gene 6000 (Qiagen). Used primers (Sigma Aldrich) are listed in Table 1.

### 2.6. Inhibitor Treatments

To analyze the influence of the proto-oncogenes MYC and NMYC as well as transcription factor NF-kB, we treated cells with MYC/NMYC inhibitor KJ-Pyr-9 (Merck) and/or dexamethasone (Dexa; Sigma Aldrich), lenalidomide (Sigma Aldrich), and PDTC (Sigma Aldrich). Cell viability was assayed using Orangu Cell Counting Solution (Cell Guidance Systems) and were performed in triplicates according to the manufacturer’s instructions. For the standard curve, 1000, 2500, 5000, 7500, and 10,000 cells and for the treatment of 3000 cells per 100 µL respective medium supplemented with 10% FCS were seeded in a 0.1% gelatin-coated 96 well-plate. After adherence of the cells (4–6 h), cell viability was measured for the standard curve and treatment was started by applying the respective inhibitor combinations. KJ-Pyr-9 was applied in the concentrations of 1, 5, 10, and 20 µM, and DMSO was used as control. Lenalidomide was used in concentrations of 30, 100, and 300 µM, and DMSO was applied as solvent control [35,36]. For the co-treatment, 10 ng/mL TNF-α, 100 µM PDTC [37,38], and 300 µM Dexa [39,40] were applied in the following conditions: (1) TNF-α, (2) TNF-α + Dexa, (3) TNF-α + PDTC, (4) TNF-α + Dexa + PDTC, (5) Dexa, (6) PDTC, (7) Dexa + PDTC. Each condition was tested with and without 10 µM KJ-Pyr-9 as well as solvent controls were carried along. After 5 days of treatment, cell viability was measured using again Orangu Cell Counting Solution (Cell Guidance Systems), and cell count was quantified using the respective standard curve. Relative survival rate was calculated by normalizing each cell count to the mean of controls for the respective cell population. Additionally, the half maximal inhibitory concentrations (IC_50_) of KJ-Pyr-9 were calculated from the log(concentration) versus normalized survival rate non-linear regression fit using Prism V5.01 software (GraphPad Software, Inc., San Diego, CA, USA).

### 2.7. Statistical Analysis

Data were raised at least in triplicate and were statistically analyzed using the Prism V5.01 software (GraphPad Software, Inc., San Diego, CA, USA). Test for normality was conducted using D’Agnostino and Pearson omnibus normality test. To evaluate differences between multiple groups, we performed unpaired *t*-test or the non-parametric Mann–Whitney test. A significance value of *p* ≤ 0.05 was considered as statistically significant. The data are presented as means ± standard error of the mean (SEM).

## 3. Results

### 3.1. Squamous Cell Carcinoma- and Adenocarcinoma-Derived Cells Depicted Stemness-like Phenotype

In this study, we aimed for the isolation of LCSC-like cells from various NSCLC tumors. Therefore, tumor material from six NSCLC patients was sampled and used for the establishment of adherently growing cells as well as cancer spheroids. Cell populations BKZ-4, BKZ-5, and BKZ-6 were isolated from male donors aged 67, 79, and 74, respectively, all suffering under squamous cell carcinomas GII (Supplementary Figure S1A–C, Table S1). The source of BKZ-7, BKZ-8, and BKZ-9 were female patients depicting invasive adenocarcinomas of the lung (Supplementary Figure S1D–F, Table S1). Analysis of clinically relevant mutations of donors of BKZ-7, -8, and -9, which were inoperable, revealed an epidermal growth factor receptor (*EGFR*) mutation for the tumor tissue of the donor of BKZ-7, with no mutation for KRAS proto-oncogene (*KRAS*), B-Raf proto-oncogene (*BRAF*), or serine/threonine kinase 11 (*STK11*). Donor of BKZ-8 did not reveal any therapeutic relevant mutation, while the tumor material of BKZ-9 donor showed mutations in the *KRAS* gene and in *STK11* (Table S2).

**Figure 1:**
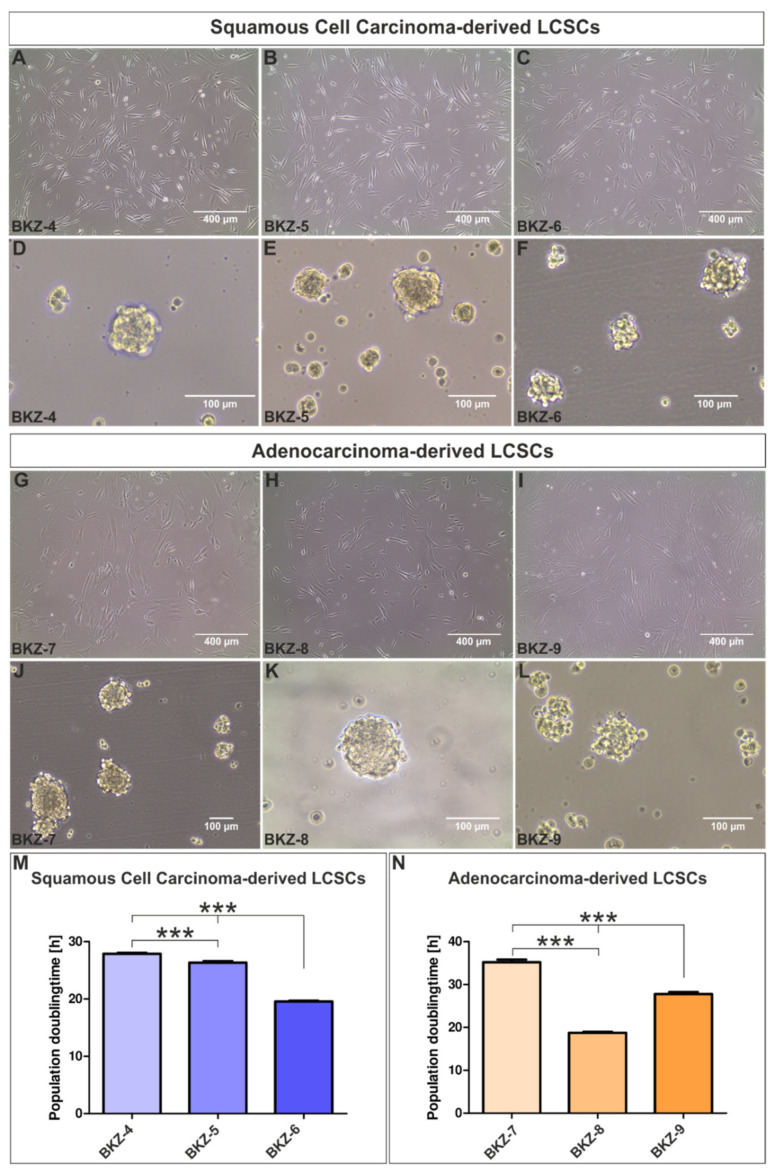
Successful isolation of squamous cell carcinoma (SCC)-derived lung cancer stem cell (LCSC)-like cell populations: (**A**) BKZ-4, (**B**) BKZ-5, and (**C**) BKZ-6. (**D–F**) Serum-free cultivation of the isolated cell populations led to the formation of free-floating cancer organoids for all three SCC-derived cell populations. Isolated adenocarcinoma (AC)-derived lung cancer stem cell-like cell populations could be also grown as (**G**–**I**) adherent culture as well as (**J**–**L**) sphere culture. (**M**) Analysis of the population doubling times of the adherent SCC-LCSCs revealed a higher population doubling time for BKZ-4 in comparison to BKZ-5 and BKZ-6, with BKZ-6 revealing the lowest doubling time. (**N**) Quantification of the population doubling times for the different AC-LCSC depicted a significantly higher population for BKZ-7 in comparison to BKZ-8 and BKZ-9. Further, population doubling time of BKZ-9 was significantly higher when compared to BKZ-8. Unpaired *t*-test (*p* ≤ 0.05). *n* = 9, *** *p* ≤ 0.001. Mean ± SEM (standard error of the mean).

Using chemically defined media, we successfully cultivated adherently growing cells with the addition of FCS as well as cancer organoids in the form of free-floating spheres in serum-free media for all six donors (Figure 1A–L). Cells cultured in 2D on the surface of tissue culture plates depicted an elongated spindle form morphology, with no obvious difference in cells derived from SCC (Figure 1A–C) in comparison to cells from AC (Figure 1G–I). Moreover, quantification of nuclear sizes revealed heterogeneity for all six populations (Supplementary Figure S2). All cell populations formed cancer organoid-like structures in serum-free media with sizes up to 100 µm (Figure 1D–F,J–L). Measurements of the population doubling time (Equations (1) and (2)) of SCC-derived cells depicted BKZ-4 as the slowest, with a mean population doubling time of 27.90 h (±0.17), whereas BKZ-6 had a doubling time of 19.56 h (±0.14) (Figure 1M). Similarly, the AC group consisted of slowly proliferating cells, such as BKZ-7, with a mean population doubling time of 35.20 h (±0.62), and those showing fast proliferation such as BKZ-8 with a population doubling of 18.76 h (±0.23) (Figure 1N).

**Figure 2:**
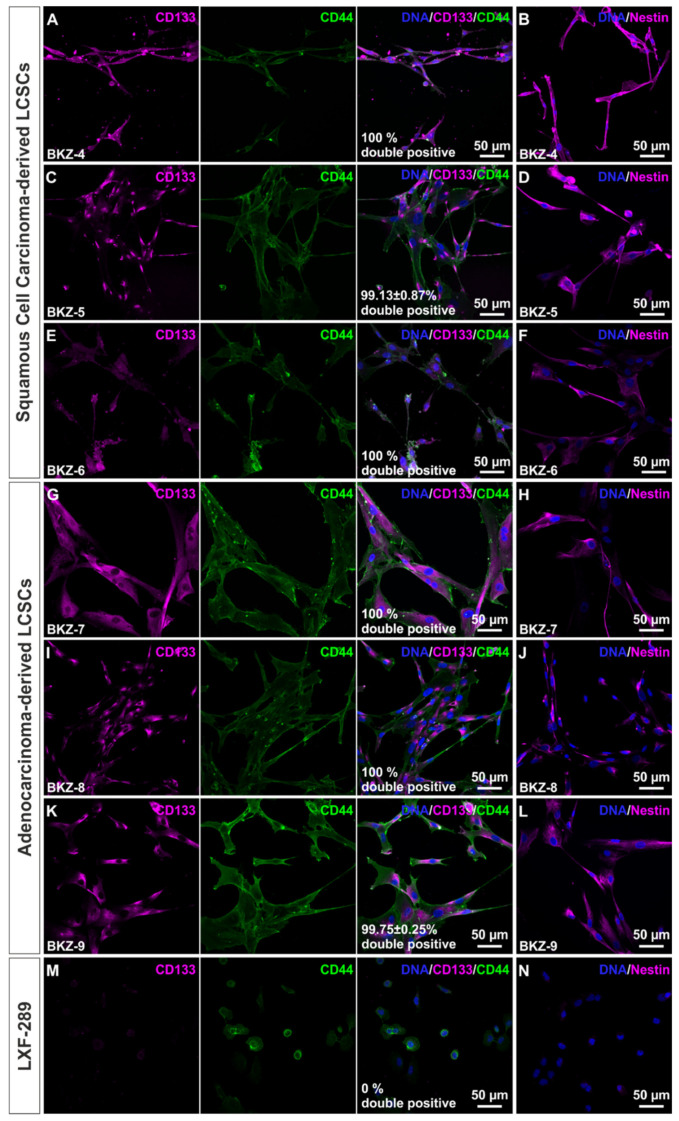
Isolated squamous cell carcinoma-derived and adenocarcinoma-derived lung cancer stem cell-like cells express higher amounts of cancer stem cell markers CD133, CD44, and Nestin in comparison to lung adenocarcinoma-derived cell line LXF-289. Immunocytochemistry revealed the presence of cancer stem cell markers CD133 and CD44 as well as the stem cell marker Nestin on protein level in (**A**–**F**) BKZ-4, BKZ-5, and BKZ-6 as well as (**G**–**L**) BKZ-7, BKZ-8, and BKZ-9. (**M**,**N**) Well-established lung adenocarcinoma-derived cell line LXF-289 did not express CD133 and Nestin and only express CD44 in low amounts in comparison to BKZ populations.

Next, we analyzed the presence of known CSC markers CD133, CD44, and Nestin on protein level and detected robust expression of CD133 and CD44 in SCC cell populations BKZ-4 (100% double-positive cells, Figure 2A) and BKZ-5 (99.13% double-positive cells, Figure 2C). Expression of CD133 and CD44 was lower but still well recognizable in BKZ-6 (100% double-positive cells Figure 2E). High expression of CD133 and CD44 was likewise observable in all three AC-derived cell populations of BKZ-7 (100% double-positive cells, Figure 2G), BKZ-8 (100% double-positive cells, Figure 2I), and BKZ-9 (99.75% double-positive cells, Figure 2K). Further, all SCC- and AC-derived cell populations likewise showed robust levels of Nestin protein (Figure 2B,D,F,H,J,L). Immunocytochemical staining of the respective CSC markers in the well-established lung adenocarcinoma-derived cell line LXF-289 revealed no expression for cancer stem cell markers CD133 and Nestin and only low expression of CD44 in comparison to LCSC-like cells (Figure 2M,N). As no expression for CD133 could be detected the percentage of CD133/CD44 double-positive cells was zero for LXF-289 cell line. Representative immunocytochemical staining for CD133 and CD44 also revealed strong expression in NSCLC-derived spheroids, as exemplarily shown in BKZ-5-derived spheres (Supplementary Figure S3). HDFs additionally served as biological negative control for immunocytochemical stainings of CSC markers (Supplementary Figure S4).

### 3.2. Tumor Necrosis Factor-α Stimulation Activated NF-κB RelA in Squamous Cell Carcinoma- and Adenocarcinoma-Derived Lung Cancer Stem Cell-like Cells

**Figure 3:**
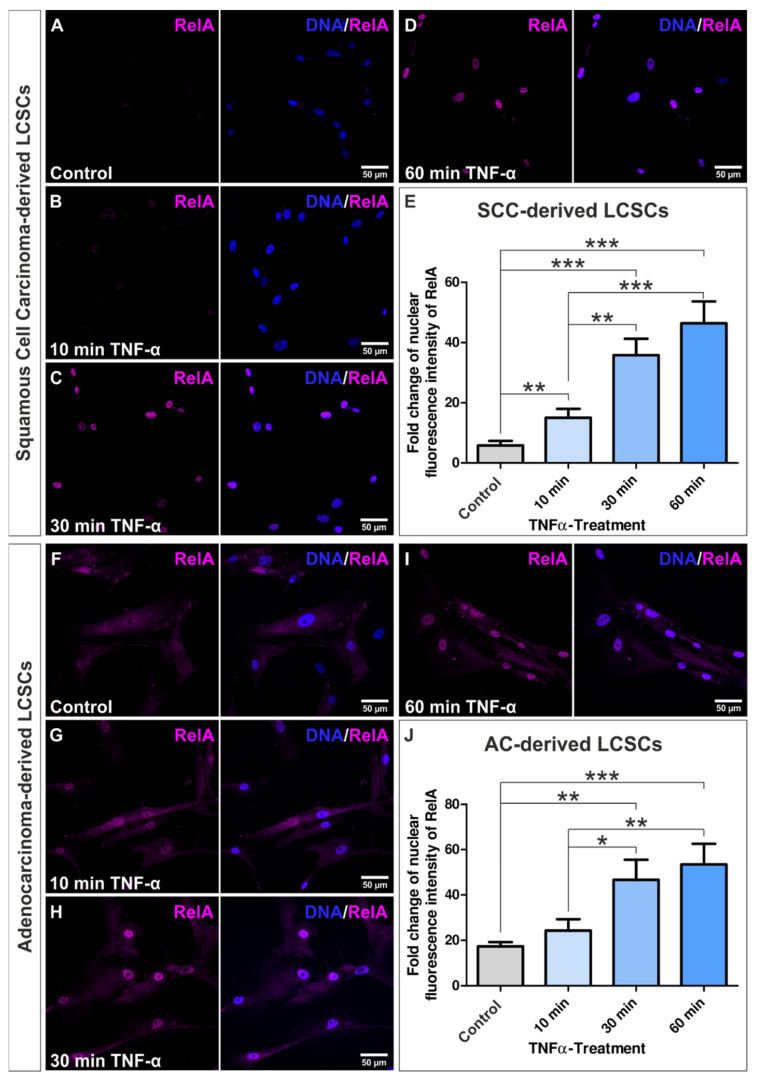
Tumor necrosis factor α (TNF-α) stimulation of non-small cell lung cancer-derived lung cancer stem cell (LCSC)-like cells activated NF-κB subunit RelA. Representative pictures of immunocytochemical staining for RelA of (**A**) untreated squamous cell carcinoma-derived BKZ-5 cells (control) and BKZ-5 cells after stimulation with TNF-α for (**B**) 10 min, (**C**) 30 min, and (**D**) 60 min. (**E**) Merged quantification of immunocytochemical assays for BKZ-4, BKZ-5, and BKZ-6 revealed a statistically significant increase of the fold change of nuclear fluorescence intensity after stimulation with TNF-α for all four time points in comparison to the control. Moreover, fold change of nuclear fluorescence intensity significantly increased with stimulation time. Accordingly, TNF-α stimulation activated NF-κB subunit RelA within adenocarcinoma (AC)-derived LCSC-like cells BKZ-7, BKZ-8, and BKZ-9. (**F**–**I**) Representative pictures of the immunocytochemical staining of RelA for AC-derived BKZ-7. (**J**) Quantification of nuclear fluorescence intensity of RelA after TNF-α stimulation for BKZ-7, BKZ-8, and BKZ-9 showed a statistically significant increase of the fold change of nuclear fluorescence intensity after stimulation with TNF-α for all four time points in comparison to the control. Unpaired *t*-test (*p* ≤ 0.05). *n* = 3, * *p* ≤ 0.05, ** *p* ≤ 0.01, *** *p* ≤ 0.001. Mean ± SEM (standard error of the mean).

Since NF-κB directs pathways linking cancer with inflammation, we analyzed expression of transactivating NF-κB subunits c-Rel, RelB, and RelA in CSC-like cells. All subunits were expressed in our established SCC- and AC-derived LCSC-like cell populations, with NF-κB RelA showing the most robust protein levels compared to RelB and cRel (Supplementary Figures S5 and S6). As NF-κB RelA was predominantly located within the cytoplasm of all six LCSC-like cells, translocation in the nucleus and thus activation of RelA was stimulated using TNF-α. Immunocytochemical analysis of the nuclear fluorescence intensity of RelA revealed its nuclear translocation in SCC-derived cells (Figure 3A–D) as well as in AC-derived cell populations (Figure 3F–I) after exposure to TNF-α. Quantification of the fold change of nuclear fluorescence intensity of all three SCC-derived cell populations showed a significant increase after only 10 min TNF-α exposure compared to the control (Equation (3), Figure 3E). Moreover, fold change of nuclear fluorescence intensity of RelA significantly increased with longer TNF-α incubation time (Figure 3E). Statistical analysis of the fold change of nuclear RelA in AC-derived cell populations showed a significant increase after TNF-α-stimulation longer than 30 min compared to the control, with a time-dependent increase from 10 min to 30 min (Figure 3J). Further analysis of the ratio of the fluorescence intensity of nuclear RelA to total RelA depicted a shift from predominantly cytoplasmic RelA with basal nuclear expression in the control towards a solely nuclear expression after 60 min of TNF-α exposure for SCC- and AC-derived cells (Supplementary Figure S7).

### 3.3. Inhibition of MYC and NMYC in Squamous Cell Carcinoma- and Adenocarcinoma-Derived Lung Cancer Stem Cell-like Cells Significantly Impaired Cell Survival

**Figure 4:**
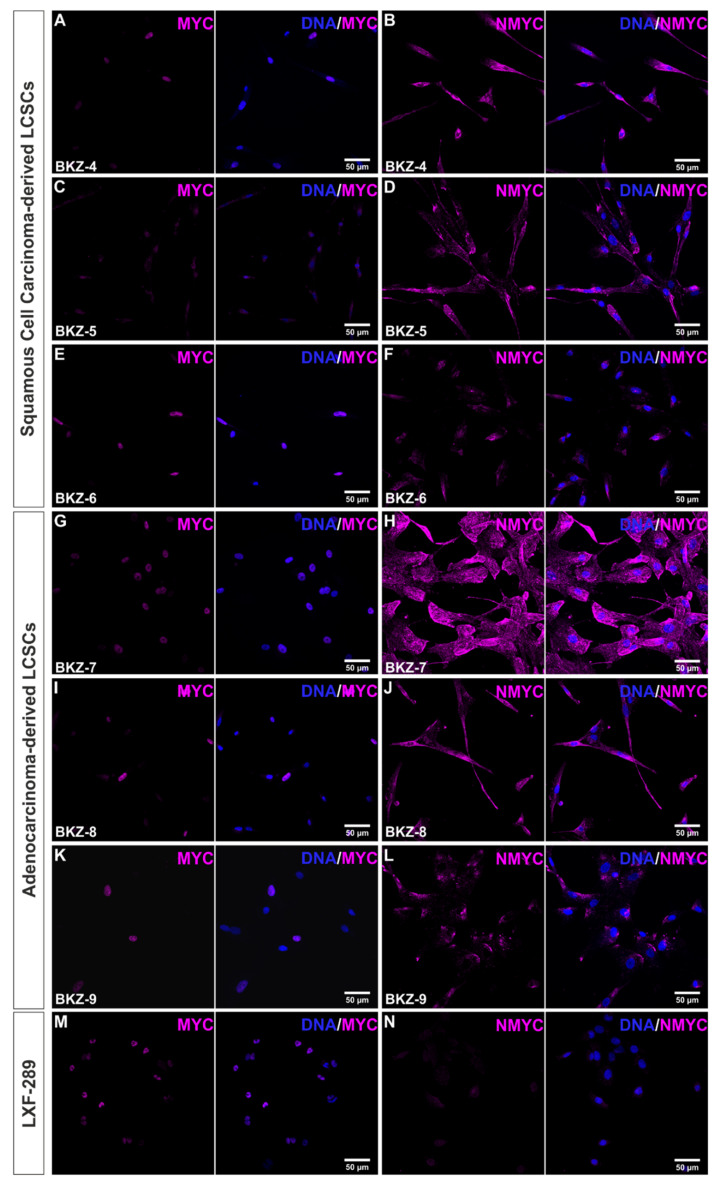
Squamous cell carcinoma- and adenocarcinoma-derived lung cancer stem cell-like cells all expressed myc proto-oncogene (MYC) and N-myc proto-oncogene (NMYC) at the protein level. Analysis of the protein expression of the oncogenes via immunocytochemical staining depicted a nuclear expression of (**A**,**C**,**E**,**G**,**I**,**K**) MYC and a predominantly cytosolic expression of (**B**,**D**,**F**,**H**,**J**,**L**) NMYC for all cell populations. Immunocytochemical analysis of the well-established lung adenocarcinoma-derived cell line LXF-289 depicted nuclear (**M**) MYC expression, as well as slight cytosolic expression of (**N**) NMYC.

The MYC family members are tightly regulated transcription factors that are responsible for the coordination of cell growth and proliferation and are thus commonly deregulated in a wide range of cancers. Immunocytochemical analysis of MYC showed nuclear localization in BKZ-4 as well as BKZ-6, with BKZ-5 only depicting slight nuclear localized MYC (Figure 4A,C,E). Next to MYC, NMYC protein was robustly expressed in all cell populations, even though expression was predominantly cytoplasmatic (Figure 4B,D,F). Analyzing MYC protein in AC-derived cells depicted nuclear protein expression in BKZ-7 and BKZ-8, but only a slight expression in BKZ-9 (Figure 4G,I,K). Of note, all three AC-cell populations were shown to robustly express mainly cytosolic NMYC (Figure 4H,J,L). Immunocytochemical analysis of the MYC and NMYC expression of the well-established lung adenocarcinoma cell line LXF-289 revealed similar expressions for MYC and a slightly lower amount NMYC in comparison to BKZ populations (Figure 4M–N). Additionally, representative immunocytochemical stainings for MYC and NMYC depicted conserved protein expression in spheroids, as exemplarily shown in BKZ-5-derived spheres (Supplementary Figure S8). Further analysis concerning MYC and NMYC protein levels using Western blot from whole-cell lysates revealed expressions within all BKZ cell populations and LXF-289 cells for MYC and NMYC, respectively (Supplementary Figure S9A). However, BKZ populations revealed additional signals for NMYC in comparison to LXF-289, possibly suggesting the presence of further isoforms of this protein in LCSC-like cells. Additionally, quantification of the relative expression levels revealed higher expression of MYC and NMYC for most of the BKZ populations in comparison to LXF-289 cells, with only BKZ-4 revealing less MYC protein. Still, some BKZ populations at least depicted up to 15-fold NMYC and 2.2-fold MYC expression. In general, NMYC expression levels varied more in AC-derived BKZ populations than in SCC-derived cells, which depicted generally lower level of NMYC. Contrarily, MYC levels varied more in SCC-derived BKZ populations in comparison to AC-derived BKZ populations (Supplementary Figure S9B). Additional investigation of the haploid copy number of MYC and NMYC of all six cell populations revealed a normal copy number (Supplementary Figure S10).

**Figure 5:**
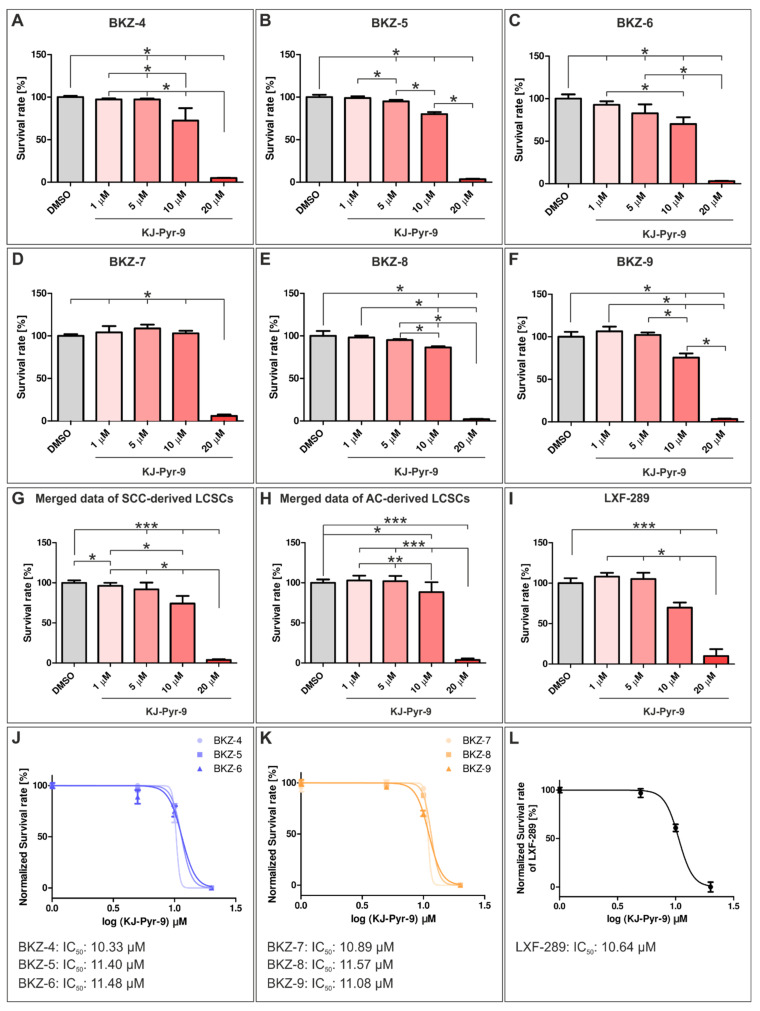
Inhibition of myc proto-oncogene (MYC) and N-myc proto-oncogene (NMYC) protein interaction with Myc-associated factor X significantly decreased survival of squamous cell carcinoma (SCC)- and adenocarcinoma (AC)-derived lung cancer stem cell (LCSC)-like cells. Metabolism was measured using Orangu, and cell count was determined by using a standard curve. Quantification of the normalized survival rate of SCC-derived (**A**) BKZ-4, (**B**) BKZ-5, and (**C**) BKZ-6 showed a significantly decreased survival after exposure to values greater than 5 µM of the MYC/NMYC inhibitor KJ-Pyr-9 in comparison to the control. Calculation of the normalized survival rate for AC-derived (**D**) BKZ-7, (**E**) BKZ-8, and (**F**) BKZ-9 showed a significantly decreased survival after exposure to 20 µM of KJ-Pyr-9 in comparison to the control. However, BKZ-8 and BKZ-9 seemed to be more sensitive in comparison to BKZ-7 as they also revealed a significantly decreased survival after the treatment with 10 µM KJ-Pyr-9. (**G**) Merged data of SCC-derived LCSC-like cells showed a significantly reduced survival after treatment with KJ-Pyr-9, with values higher than 5 µM for and (**J**) half maximal inhibitory concentration (IC_50_) values between 10.33 and 11.48 µM. (**H**) Quantification of merged data of AC-derived LCSC-like cell populations showed significantly reduced survival upon KJ-Pyr-9 values higher than 10 µM. (**K**) IC_50_ values ranged between 10.89 µM and 11.57 µM KJ-Pyr-9 for AC-derived LCSC-like cells. (**I**) Quantification of the survival of lung adenocarcinoma cell line LXF-289 showed significantly reduced survival after exposure to 10 µM KJ-Pyr-9 with an (**L**) IC_50_ of 10.64 µM. Non-parametric Mann–Whitney test (**A**–**G**,**I**, *p* ≤ 0.05). Unpaired *t*-test (**H**, *p* ≤ 0.05). *n* = 3, * *p* ≤ 0.05, ** *p* ≤ 0.01, *** *p* ≤ 0.001. Mean ± SEM (standard error of the mean).

On the basis of the consistent expression of MYC and NMYC in all NSCLC-derived cells, we examined the influence of the small molecule KJ-Pyr-9, an inhibitor of the protein–protein interaction of MYC/NMYC with MAX. Usage of KJ-Pyr-9 doses higher than 5 µM significantly decreased survival rates for BKZ-4, BKZ-5, and BKZ-6. However, for BKZ-6, even a concentration of 1 µM KJ-Pyr-9 impaired survival. Treatment with 10 µM KJ-Pyr-9 led to a significant decrease of the survival rates of BKZ-4 with 72.42% (±8.43), BKZ-5 with 80.01% (±1.4), and BKZ-6 with 70.23% (±4.62). Nevertheless, especially the treatment with 20 µM KJ-Pyr-9 showed a potential therapeutically relevant effect on survival of BKZ-4, -5, and -6, as survival rates were impaired to 4.92% (±0.16), 3.58% (±0.32), and 3.09% (±0.19), respectively (Figure 5A–C). Statistical analysis of merged data of all three SCC populations showed a highly significant effect of KJ-Pyr-9 values greater than 5 µM on cell survival, even though only 20 µM of KJ-Pyr-9-treatment led a reduction of more than 95% survival (Figure 5G). Calculation of the half maximal inhibitory concentration (IC_50_) of KJ-Pyr-9 revealed IC_50_ values of 10.33 µM for BKZ-4, 11.40 µM for BKZ-5, and 11.48 µM for BKZ-6 (Figure 5J). In accordance with SCC-derived cells, KJ-Pyr-9-treatment of AC-derived cells showed similar results. Nevertheless, for AC-derived cells, only concentrations greater than 10 µM KJ-Pyr-9 revealed a significant effect on cell survival compared to control. Again, usage of 20 µM KJ-Pyr-9 reduced cell survival significantly for BKZ-7 to 5.98% (±0.93), BKZ-8 to 2.02% (±0.33), and BKZ-9 to 3.36% (±0.28) (Figure 5D–F). Analysis of the merged data of all three AC-derived LCSC-like cells showed significant reductions of cell survival after the exposure to KJ-Pyr-9 concentrations greater than 10 µM with survival of 88.38% (±4.11). However, only application of 20 µM KJ-Pyr-9 impaired cell survival in a highly significant way, with final survival of only 3.79% (±0.65) (Figure 5H). Analysis of the half maximal inhibitory concentration (IC_50_) of KJ-Pyr-9 depicted IC_50_ values of 10.89 µM for BKZ-7, 11.57 µM for BKZ-8, and 11.08 µM for BKZ-9 (Figure 5K). Investigation of the influence of KJ-Pyr-9 on LXF-289 cells revealed significant reductions in cell survival after exposure to 10 µM KJ-Pyr-9 with 69.78% (±3.62) and 20 µM KJ-Pyr-9 with 9.877 (±4.94) survival left (Figure 5I). Calculated IC_50_ was 10.64 µM (Figure 5L). Interestingly, 24 h treatment of NSCLC-derived cell populations with 10 or 20 µM KJ-Pyr-9 led to heterogeneous nuclear localization of MYC for SCC- and AC-derived cell populations (Supplementary Figure S11).

**Figure 6:**
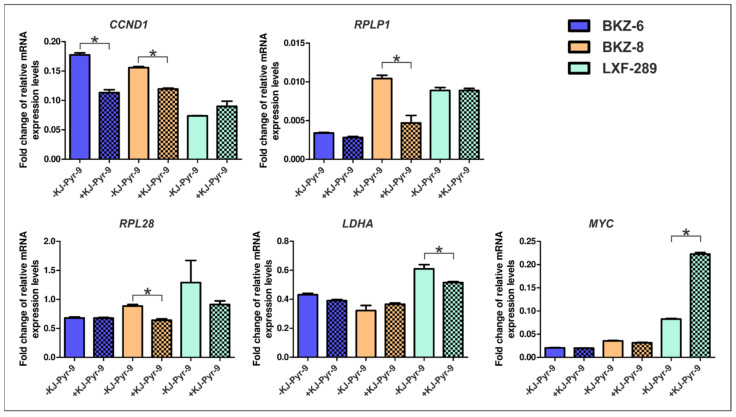
Myc proto-oncogene (MYC)-regulated target genes in lung cancer stem cell (LCSC)-like cells. Quantitative polymerase chain reaction of different target genes of MYC revealed a significant reduction of Cyclin D1 (*CCND1*) after the application of 20 µM KJ-Pyr-9 for BKZ-6 and BKZ-8 as representative cell populations for squamous cell carcinoma- and adenocarcinoma-derived LCSC-like cells. Further, BKZ-8 exhibited significantly reduced mRNA expressions of ribosomal protein lateral stalk subunit P1 (*RPLP1*) and ribosomal protein L28 (*RPL28*). Additionally, lung adenocarcinoma cell line LXF-289 showed a significantly decreased mRNA level of lactate dehydrogenase A (*LDHA*) and a significantly increase in *MYC* expression after KJ-Pyr-9 treatment. Non-parametric Mann–Whitney-test (*p* ≤ 0.05). *n* = 3, * *p* ≤ 0.05. Mean ± SEM (standard error of the mean).

Analysis of potential target genes of MYC involved in the KJ-Pyr-9-induced survival decrease revealed a decrease of cyclin D1 (*CCND1*) after 24 h treatment with 20 µM KJ-Pyr-9 for BKZ-6 and BKZ-8 as representative cell populations for squamous cell carcinoma- and adenocarcinoma-derived LCSC-like cells. Additionally, BKZ-8 exhibited significantly reduced mRNA expression levels of ribosomal protein lateral stalk subunit P1 (*RPLP1*) and ribosomal protein L28 (*RPL28*), both involved in ribosomal biosynthesis. Not regulated in LCSC-like cells but regulated in the lung adenocarcinoma cell line LXF-289 were lactate dehydrogenase A (*LDHA*) and *MYC* mRNA expressions, as *LDHA* was significantly reduced and *MYC* significantly increased after KJ-Pyr-9 application (Figure 6). Further tested but not regulated MYC target genes were cyclin D3 (*CCND3*), ribosomal protein L5 (*RPL5*), and ribosomal protein L14 (*RPL14*) (Supplementary Figure S12). Additional analysis of the number of senescent cells after treatment with 20 µM KJ-Pyr-9 for 24 h led to a significant increase in SCC-derived senescent cells, but not in AC-derived senescent cells (Supplementary Figure S13).

### 3.4. Inhibition of NF-κB Signaling Decreased Survival of Squamous Cell Carcinoma- and Adenocarcinoma-Derived Lung Cancer Stem Cell-like Cells

**Figure 7:**
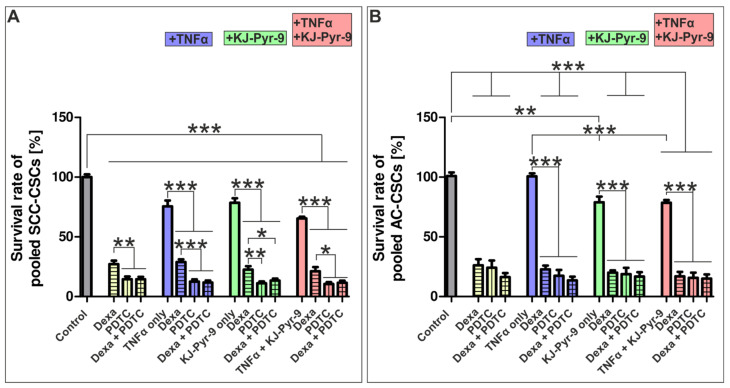
Co-inhibition of MYC and NF-κB signaling did not synergistically affect survival of NSCLC-derived LCSC-like cells. To analyze possible synergistic effects of MYC and NF-κB signaling inhibition on cell survival, we treated cells with dexamethasone (Dexa; 300 µM), PDTC (100 µM), TNF-α (10 ng/mL), and KJ-Pyr-9 (10 µM), and cellular viability was analyzed using Orangu. (**A**) Statistical analysis of normalized survival rates of SCC-derived LCSC-like cells revealed a significantly reduced survival upon all treatment combinations but did not show any synergistically effect of the different reagents. There was no difference in cell survival upon the usages of dexamethasone and PDTC in combination with TNF-α, KJ-Pyr-9, or TNF-α with KJ-Pyr-9 together. (**B**) Quantification of the normalized AC-derived cell survival also revealed no synergistical effect of inhibition of MYC and NF-κB signaling. However, TNF-α alone did not influence cell survival of AC-derived cells in contrast to SCC-derived ones. Unpaired *t*-test (*p* ≤ 0.05). *n* = 3, * *p* ≤ 0.05, ** *p* ≤ 0.01, *** *p* ≤ 0.001. Mean ± SEM (standard error of the mean).

Next to the influence of MYC/NMYC inhibition, we investigated NSCLC-derived cell survival after inhibition of NF-κB signaling utilizing dexamethasone and PDTC. Additionally, the influence of possible synergistic effects on the impairment of cell survival was determined by utilizing different combinations of these inhibitors with TNF-α and KJ-Pyr-9. Here, treatment with dexamethasone (300 µM) and PDTC (100 µM) led to significant reductions of SCC- and AC-derived cell survival (Figure 7A,B). On the contrary, application of the NF-κB inhibitor lenalidomide only slightly impaired cell survival of SCC-derived cells with nearly no effect on AC-derived LCSC-like cells (Supplementary Figure S14). PDTC-treatment in SCC-derived LCSC-like cells led to a significantly elevated reduction with only 14.60% (±2.17) cell survival left in comparison to dexamethasone with 27.17% (±5.17) survival (Figure 7A). This effect was not detectable for AC-derived LCSC-like cells, as 26.24% (±5.12) survival was observable after dexamethasone treatment and still 24.16% (±6.04) survival after the exposure to PDTC (Figure 7B). No additional effect on survival reduction of NSCLC-derived cells was detectable after application of dexamethasone and PDTC, even though a tendency could be seen for AC-derived LCSC-like cells (Figure 7A,B). In addition, treatment of SCC- and AC-derived LCSC-like cells with PDTC resulted in a significant increase in senescence in comparison to the control (Supplementary Figure S13). Interestingly, nuclear RelA localization was only slightly affected by PDTC-treatment for 24 h, although a tendency showing a decrease in the ratio of nuclear RelA to total RelA was observable after exposure of LCSC-like cells to PDTC compared to control (Supplementary Figure S15).

Analysis of the influence of TNF-α (10 ng/mL) on NSCLC-derived LCSC-like cells exhibited a survival-decreasing effect only on SCC-derived LCSC-like cells (75.51% (±4.92) survival), but no effect for AC-derived cells. Nevertheless, no synergistic depletion of cellular survival of NSCLC-derived LCSC-like cells was detectable by co-treatments using TNF-α with dexamethasone and/or PDTC together. Accordingly, combined MYC/NMYC and NF-κB inhibition using 10 µM KJ-Pyr-9 with dexamethasone and/or PDTC did not result in synergistic reductions in survival of NSCLC-derived LCSC-like cells. Of note, MYC/NMYC inhibition using only 10 µM KJ-Pyr-9 alone decreased survival of SCC- and AC-derived LCSC-like cells significantly. The use of TNF-α, KJ-Pyr-9, dexamethasone, and PDTC in parallel did not impair NSCLC-derived LCSC-like cell survival synergistically (Figure 7A,B). In summary, the inhibition of MYC/NMYC signaling using KJ-Pyr-9 as well as the inhibition of NF-κB signaling utilizing dexamethasone or PDTC significantly reduced survival of SCC- and AC-derived LCSC-like cells with no synergistic effects (Table 2).

## 4. Discussion

In this study, we present six novel LCSC-like cell populations derived from three SCC and three AC of the lung as promising in vitro models for LCSC-like cells. Usage of low doses of the MYC signaling inhibitor KJ-Pyr-9 led to a significant depletion in survival of SCC- as well as AC-derived LCSC-like cells, representing the impairment of protein–protein interaction of MYC/NMYC, with MAX as a promising target in treating NCSLC, particularly by engaging MYC high-expressing LCSC. One possibly relevant MYC target gene, which we detected to be regulated by KJ-Pyr-9, was cyclin D1. Nevertheless, the whole picture of the underlying working mechanism still remains elusive.

Cancer stem cells are defined by the expression of a set of different markers such as CD133 [41], CD44 [42], and Nestin [43]. In primary NSCLC cell lines, Chen and colleagues demonstrated that CD133^+^ cells displayed higher ability for self-renewal and tumor initiation, as well as higher resistance to chemotherapy in comparison to CD133^-^ cells [44]. This in vivo tumorigenicity and the correlation of the expression of stemness-related genes of CD133^+^ cells was confirmed additionally by Tirino and Huang [45,46]. Further, CD44 expression was shown to correlate with stem cell-like properties such as chemotherapy resistance, enhanced spheroid formation and tumor-initiating capacities [47,48]. Moreover, CD44 expression was demonstrated to be linked to drug resistance [49], NSCLC occurrence, and metastasis [50,51]. Double-positive CD133^+^/CD44^+^ primary AC-derived lung cancer cells revealed higher colony formation units than CD133^-^/CD44^-^ ones. Further, Wang and colleagues depicted that IL6 pretreatment of CD133^+^/CD44^+^ cells assisted in entering into the cell cycle in quiescent lung cancer stem cells and significantly increased chemosensitivity [52]. Nestin initially served as stem cell marker especially for the central nervous system [53], although its role as CSC marker became more prominent in recent years. Regarding NSCLC, a knockdown of Nestin protein using short hairpin RNA not only decreased proliferation but also affected migration, invasion, and sphere formation of AC-derived cells [54]. This stands in accordance with Liu and coworkers, who indicated that a CRISPR/Cas9-mediated knockout of Nestin led to reduced proliferation, invasion, and colony formation of H1299 and A549 cell lines [55]. Even though, some studies of NSCLC-derived cancer stem cells exist, most of them are based on sorted well-established cell lines and not on primary ones. Thus, the herein presented co-expression of CSC markers CD133 and CD44, as well as the expression of Nestin in the novel BKZ-4, -5, -6, -7, -8, and -9 cell populations, strongly suggests their LCSC-like character and represents these as promising new in vitro models for studying NSCLC-derived stem cells. Additionally, immunocytochemical investigation of the well-established lung adenocarcinoma cell line LXF-289 revealed no expression for CD133 and Nestin and lower expression of CD44, underpinning the presence of a CSC phenotype in the here-presented BKZ cell populations. Tumor spheroid formation of all six BKZ cell populations further substantiated LCSC properties, as cell populations with tumor-sphere-forming-properties are constantly shown to form tumors in xenograft models of LCSC-like cells [56].

Myc family members are key regulators of cell proliferation, self-renewal, and differentiation and are known to play an important role in tumor initiation and progression [57]. Recently, we reported a significant survival-decreasing effect in primary human colon cancer stem-like cells by the inhibition of MYC signaling using KJ-Pyr-9 [29]. While KJ-Pyr-9 is a small molecule with highest activity in MYC inhibition, it is interesting to note that recent efforts in drug development led to new derivates with similar activity but higher solubility and better stability [58]. Regarding NSCLC, the working group around Tao reported that viability, self-renewal, and invasion of A549-derived LCSC-like cells was decreased by affecting MYC signaling [18]. Targeting MYC also enhanced chemotherapeutic efficacy of cisplatin in NSCLC cells [59]. In accordance with these results, we here observed a high protein amount of MYC and NMYC in all six NSCLC-derived LCSC-like cells, further underlining their stem-like characteristics. Notably, inhibition of MYC by application of 20 µM small molecule KJ-Pyr-9 [28] resulted in strong inhibition of cell survival in LCSC-like cells, while concentration of up to 10 μM KJ-Pyr-9 led to a slight yet significant decrease in survival. Calculation of the half maximal inhibitory concentrations (IC_50_) revealed IC_50_ values between 10.33 and 11.57 µM KJ-Pyr-9 for the here-established LCSC-like cells. Accordingly, recombinant MYC–MAX–DNA interaction was reported to be inhibited with an IC_50_ of approximately 10 and 30 μM [28,60]. On the contrary, KJ-Pyr-9 was described to have no effect on MYC–MAX in an SPR assay up to 10 μM, with the reason for this discrepancy being unclear [61]. Investigation of lung adenocarcinoma cell line LXF-289 revealed similar results concerning the influence of MYC inhibition on cell survival, suggesting a conserved working mechanism on lung adenocarcinoma cells with MYC expression. Nevertheless, MYC and NMYC expression of LXF-289 were principally lower in comparison to BKZ populations, except for BKZ-4 for MYC protein. Furthermore, LCSC-like cells revealed additional signals within the NMYC blot, suggesting the presence of further isoforms of this protein in LCSC-like cells in comparison to LXF-289. However, the characteristics and role of these possible new isoforms have to be investigated in future studies. To gain first insights into the working mechanism of KJ-Pyr-9-induced survival decrease in MYC expressing lung cancer cells, we tested for alterations in mRNA levels of several known MYC target genes, such as *CCND1*, *CCND3*, and *MYC* itself. Further, we recently published high expressions of genes involved in ribosomal biosynthesis in different CSC-like populations, including the here-presented BKZ-7, BKZ-8, and BKZ-9 (there referred to as LCSC_a, LCSC_b, and LCSC_c [62]). Here, we focused on known MYC target genes, such as *RPLP1*, *RPL5*, *RPL14*, *RPL28*, and *LDHA* [63,64,65,66,67]. Quantification revealed significant reductions in *CCND1* mRNA levels for the representative LCSC-like cell populations BKZ-6 and BKZ-8 after KJ-Pyr-9 application, possibly explaining the survival-decreasing effects. This stands in line with studies of Zhou et al. and Chen et al. that reported MYC-dependent cell cycle regulation in NSCLC cell lines [68,69]. Furthermore, *RPLP1* and *RPL28* expression in BKZ-8 was affected by KJ-Pyr-9 application, suggesting an involvement of MYC signaling in ribosomal biosynthesis of AC-derived LCSC-like cells, as already shown for several cancer types (reviewed in [70,71]). However, lung adenocarcinoma cell line LXF-289 and SCC-derived LCSC-like cell population BKZ-6 did not reveal differences in the investigated genes involved in ribosomal biosynthesis, highlighting a cell line-specific response. These variances could also be seen in the response of *LDHA*, as it was only downregulated in LXF-289 cells but not in BKZ-6 or BKZ-8. In pancreatic and prostate cancer, a correlation of MYC with LDHA expression was detected and linked to the regulation of aerobic glycolysis while promoting tumor progression and decreasing apoptosis [67,72]. Thus, downregulation of *LDHA* may be one cause for KJ-Pyr-9-mediated decrease in cell survival of LXF-289. Nevertheless, this effect could not be detected in the two representative BKZ populations, suggesting a difference between the here-established LCSC-like cells and the LXF-289 cell line. Further, the in 1995 established lung adenocarcinoma-derived cell line LXF-289 [30] showed atypical effects upon MYC inhibition, as mRNA levels of the transcript were increased by KJ-Pyr-9 treatment. Generally, it is postulated that MYC creates a positive auto-regulatory circuit, which is essential for sustaining mutual high expression in tumor cells [73]. However, the data presented in this manuscript revealed a more complex mechanism involved in the regulation of MYC signaling, suggesting the need for large-scale target gene analysis to fully understand the signaling cascade. In conclusion, our observations strongly emphasized MYC inhibition as an auspicious therapy for treating NSCLC by targeting cyclin D1 expression in LCSC-like cells, while the whole underlying mechanism still remains unclear.

Next to MYC, NF-κB signaling is broadly described to be involved in multiple steps of lung carcinogenesis, to mediate therapy resistance [74], and to be active in lymph node metastasis [27], providing an important linkage between the pathogenesis of pulmonary inflammation and lung cancer [75]. Accordingly, the presence of NSCLC cells positive for NF-κB RelA was reported to be correlated with shorter overall survival time, suggesting RelA expression as a prognostic factor for NSCLC [27,76] (reviewed in [77]). Regarding its role in LCSCs, NF-κB was also reported to be highly associated with the CSC gene expression signature of NSCLC cells [10]. Depletion of NF-κB RelA utilizing the kinase inhibitor BMS-345541 effectively reduced stemness and EMT markers, self-renewal, and migratory properties of LCSCs [78], suggesting NF-κB as a promising target for CSC depletion in NSCLC (reviewed in [77]). Accordingly, we observed RelA protein in high amounts in SCC- and AC-derived LCSC-like cells and thus conclude that RelA expression seems to be a unifying factor of all NSCLC-derived cell populations described here. Application of TNF-α led to nuclear translocation and thus to the activation of RelA in all six LCSC-like cells. TNF-α-mediated signaling in NSCLC cell lines was shown to be favorable for cancer initiation, as TNF-α-induced NF-κB signaling led to the protection from cell death [79] and was shown to have a metastasis-promoting effect associated with tumor recurrence and drug resistance [80]. On the other hand, TNF-α expression was reported to be unfavorable as it was involved in necroptosis of NSCLC cells [81]. Additionally, there is a concept of pro-tumor inflammation being present in cancer [82]. Regarding NSCLC, anti-inflammatory therapy targeting the interleukin-1β innate immunity pathway was shown to significantly reduce lung cancer mortality [83]. In the present study, we investigated this concept of pro-inflammatory cytokines as growth factors for CSCs by using TNF-α, but could not detect any increase in cell number in comparison to control. Accordingly, analysis of cell survival after TNF-α-stimulation revealed no change in survival of AC-derived LCSC-like cells, but a significant reduction in survival of SCC-derived LCSC-like cells, reflecting differences in the dependence on TNF-α/NF-κB-mediated pathways between NSCLC-derived LCSC-like cells.

Inhibitors of pro-inflammatory signaling such as dexamethasone, lenalidomide, or PDTC are known to influence the production of cytokines and growth factors, which in turn enhances the immune response against tumor cells and inhibits tumor angiogenesis [84]. Even though proliferation of some NSCLC cell lines have been shown to be affected by lenalidomide [85], we here observed fivefold higher doses to marginally impair survival of NSCLC-derived LCSC-like cells in comparison to the study around Kim and colleagues [85]. This kind of lenalidomide resistance possibly highlights their enrichment for stem-like properties. Lenalidomide was shown to affect NF-kB-induced apoptosis by modulating the production of cytokines and growth factors such as TNF-α and insulin-like growth factor–1 [86], thus acting more upstream in comparison to PDTC and dexamethasone. Hence, the here-presented LCSC-like cells potentially evade the immunomodulatory effects of lenalidomide by a mechanism more downstream of the respective signaling pathway. Nevertheless, underlying mechanisms have to be clarified. Contrarily, application of the immunomodulatory synthetic glucocorticoid dexamethasone resulted in a more elevated reduction in cell survival of NSCLC-derived LCSC-like cells. Dexamethasone was shown to interfere with NF-κB activation and reduced TNF-α production [87,88]. In NSCLC cells it was shown to inhibit TGF-β1-induced migration as well as EMT via the AKT/ERK signaling pathways [89], suggesting a modulatory effect on CSC properties. Nevertheless, dexamethasone is also suggested to impair chemotherapy efficacy, as co-administration was shown to decrease chemosensitivity in NSCLC xenograft models [90]. Next to dexamethasone, PDTC is commonly known to inhibit the IkBα degradation and p65 nuclear import [91], and was likewise shown to reduce survival of NSCLC-derived LCSC-like cells in the present study. Accordingly, Zhang and coworkers demonstrated that PDTC regulates metastasis of A549 cells in co-culture with lymphatic endothelial cells, possibly representing a link between NF-κB and the lymphocytic metastasis of NSCLC cells [92]. In this study, PDTC treatment was more effective in decreasing cell survival of SCC-derived LCSC-like cells in comparison to high doses of dexamethasone. However, this difference could not be detected in AC-derived LCSC-like cells, suggesting a more chemoresistant phenotype compared to SCC-derived cells. Two of three parental tissues of AC-derived LCSC-like cells revealed clinically relevant mutations, one for *EGFR* and one for *KRAS* and *STK11*, possibly explaining the observed reduction in sensitivity for PDTC. However, SCC-derived cells were not tested for potential mutations, as no clinical relevance was existent. Generally, co-treatment with PDTC and dexamethasone, and especially application of KJ-Pyr-9 seem to be more effective in targeting NSCLC-derived LCSC-like cells in comparison to various standard chemotherapeutics such as cisplatin, docetaxel, pemetrexed, paclitaxel or vinorelbine as investigated by Herreros-Pomares and colleagues. Those chemotherapeutics were shown to impair CSC survival only marginally with 83.5%, 86.9%, 76.2%, 68.2%, and 56.9% cells alive after treatment, respectively. More effective, but still not as effective as KJ-Pyr-9 and co-treatment with PDTC and dexamethasone, was salinomycin with 21.7% survival left [93] (Table 2). Salinomycin was already identified as highly active drug, reducing breast CSCs more than 100-fold in comparison to standard drug treatment with paclitaxel [94]. Further, it was shown to inhibit NF-κB activity in prostate cancer cells, which was reported by Ketola and coworkers [95]. Thus, we conclude that targeting of the NF-kB pathway with drugs such as PDTC or salinomycin might be a new route for post-surgical treatment of NSCLC. Nevertheless, MYC seems to also play a pivotal role, as its expression was also impaired by salinomycin [95]. Interestingly, PDTC was reported to induce cytotoxic effects against SCLC cells by suppressing MYC expression and inducing S phase arrest [96], suggesting a role of PDTC in MYC modulation in NSCLC cells. Still, direct inhibition of MYC/NMYC using low doses of KJ-Pyr-9 was shown to be more effective than PDTC-treatment for both SCC- and AC-derived LCSC-like cells. Additionally, no synergistic effect of the inhibition of NF-κB and MYC signaling was detectable in NSCLC-derived LCSC-like cells, suggesting either inhibition of MYC signaling or NF-κB signaling to inhibit survival of LCSCs without a cross-coupling between the two pathways.

In summary, we successfully isolated six novel NSCLC-derived lung cancer stem-like cells as promising in vitro models to further study CSC–driven tumor growth, treatment resistance, and cancer relapse. Expression of the prominent CSC markers CD133, CD44, and Nestin as well as successful formation of spherical cancer organoids confirmed a CSC-like phenotype. Additionally, all three AC- as well as SCC-derived LCSC-like cells expressed proto-oncogenes MYC and NMYC, further emphasizing their stem-like characteristics. Application of inhibitors of NF-κB and MYC signaling led to significant reductions in survival of both AC- and SCC-derived cells. Nevertheless, inhibition of MYC/NMYC using KJ-Pyr-9 was observed to impair LCSC-like cells most effectively, suggesting MYC signaling as a possible object for targeting LCSC. Even though MYC signaling could be identified to play a crucial role in LCSC-like survival, underlying mechanisms involved in this signaling pathway still remain unclear, and MYC-inhibiting drugs have to be clinically approved to be able to use them against NSCLC. In contrast, dexamethasone and the PDTC derivative zinc diethyldithiocarbamate are already in clinical use and thus more rapidly applicable against NSCLC. Overall, both signaling pathways are promising targets in NSCLC-derived LCSC-like cells, but molecular signaling needs further investigation.

## Figures and Tables

**Table 1:** Primer sequences for quantitative polymerase chain reaction.

Target Gene	Sequence 5′–3′
*NMYC* (genomic)	CGCAAAAGCCACCTCTCATTA
Rev-*NMYC* (genomic)	TCCAGCAGATGCCACATAAGG
*MYC* (genomic)	AAAAGTGGGCGGCTGGATAC
Rev-*MYC* (genomic)	AGGGATGGGAGGAAACGCTA
Syndecan 4 (genomic)	CAGGGTCTGGGAGCCAAGT
Rev-Syndecan 4 (genomic)	GCACAGTGCTGGACATTGACA
Glyceraldehyde-3-phosphate dehydrogenase (*GAPDH*) (genomic)	AGACTGGCTCTTAAAAAGTGCAGG
Rev-*GAPDH* (genomic)	TGCTGTAGCCAAATTCGTTGTC
Beta-actin (*ACTB*)	CTTCGCGGGCGACGAT
Rev-*ACTB*	CCACATAGGAATCCTTCTGACC
Cyclin D1 (*CCND1*)	ATGCCAACCTCCTCAACGAC
Rev-*CCND1*	TCTGTTCCTCGCAGACCTCC
Cyclin D3 (*CCND3*)	ACTGGCACTGAAGTGGACTG
Rev-*CCND3*	GGGCTACAGGTGTATGGCTG
Lactate dehydrogenase A (*LDHA*)	CTTGACCTACGTGGCTTGGA
Rev-*LDHA*	CCAGCCTTTCCCCCATTAGG
Ribosomal protein L5 (*RPL5*)	CAGCGTATGCACACGAACTG
Rev-*RPL5*	ACCTATTGAGAAGCCTGCGG
Ribosomal protein L14 (*RPL14*)	TTGGACCTCATGCCGGAAAA
Rev-*RPL14*	GCACTGTGCGGAAACTTGAG
Ribosomal protein L28 (*RPL28*)	CTCTTTCCGTCTCAGGTCGC
Rev-*RPL28*	TCTTGCGGTGAATCAGTCCG
Ribosomal protein P1 (*RPLP1*)	TGAAAACTGCACTGGGGTGG
Rev-*RPLP1*	AGGGTAAATACCCAGGAGGCT
*GAPDH* *Rev-GAPDH*	CATGAGAAGTATGACAACAGCCT AGTCCTTCCACGATACCAAAGT
*MYC*	GGCACTTTGCACTGGAACTT
Rev-*MYC*	AGGCTGCTGGTTTTCCACTA

**Table 2:** Survival rates of squamous cell carcinoma (SCC)- and adenocarcinoma (AD)-derived lung cancer stem cell (LCSC)-like cells after the application of different NF-κB inhibitors and the MYC inhibitor KJ-Pyr-9.

Treatment	Concentration	Target	Survival Rate of SCC-Derived LCSC-Like Cells	Survival Rate of AD-Derived LCSC-Like Cells
KJ-Pyr-9	1 µM	MYC	96.35% (±1.23)	103.0% (±1.99)
KJ-Pyr-9	5 µM	MYC	91.77% (±2.86)	102.1% (±2.16)
KJ-Pyr-9	10 µM	MYC	74.22% (±3.17)	88.38% (±4.11)
KJ-Pyr-9	20 µM	MYC	3.86% (±0.30)	3.80% (±0.65)
PDTC ^1^	100 µM	NF-κB	14.60% (±2.17)	24.16% (±6.04)
Dexa ^2^	300 µM	NF-κB	27.17% (±5.17)	26.24% (±5.12)
PDTC + Dexa	100 µM/300 µM	NF-κB	14.61% (±1.91)	16.33% (±3.29)
KJ-Pyr-9 + PDTC	10 µM/100 µM	MYC/NF-κB	11.18% (±1.48)	18.84% (±5.25)
KJ-Pyr-9 + Dexa	10 µM/300 µM	MYC/NF-κB	22.53% (±2.95)	20.09% (±1.90)
KJ-Pyr-9 + PDTC + Dexa	10 µM/100 µM/300 µM	MYC/NF-κB	13.40% (±1.60)	16.84% (±3.61)

^1^ PDTC = pyrrolidinedithiocarbamate; ^2^ Dexa = dexamethasone.

## Supplementary Materials

The following are available online at https://www.mdpi.com/article/10.3390/cells10051024/s1, Figure S1: Clinical imaging of the different donors of the six isolated lung cancer stem cell-like cell populations. Figure S2. SCC- and AC-derived LCSC-like cells were heterogeneous in size. Figure S3. BKZ-5-derived spheres expressed cancer stem cell markers Prominin-1 (CD133) and CD44-antigen (CD44). Figure S4. Immunocytochemical staining of cancer stem cell markers in adult human dermal fibroblasts (HDF). Figure S5. Squamous cell carcinoma-derived lung cancer stem cell-like cells BKZ-4, BKZ-5, and BKZ-6 all expressed NF-κB subunits RelA, RelB, and cRel. Figure S6. Adenocarcinoma-derived lung cancer stem cell-like cells all predominantly expressed NF-κB subunits RelA, RelB, and cRel. Figure S7. Stimulation with tumor necrosis factor α (TNF-α) shifted cytoplasmic NF-κB RelA into the nucleus of squamous cell carcinoma- and adenocarcinoma-derived lung cancer stem-like cells. Figure S8. BKZ-5-derived spheres expressed myc proto-oncogene (MYC) and slightly N-myc proto-oncogene (NMYC). Figure S9. Western blot analysis of the protein levels of NMYC and MYC within BKZ populations and LXF-289 cells. Figure S10. Squamous cell carcinoma (SCC)- and adenocarcinoma (AC)-derived LCSC-like cells all revealed a normal copy number for myc proto-oncogene (MYC) and N-myc proto-oncogene (NMYC). Figure S11. Treatment of non-small cell lung cancer-derived cell populations with KJ-Pyr-9 led to heterogeneous nuclear localization of myc proto-oncogene (MYC). Figure S12: Analysis of putative MYC-target genes in lung cancer stem cell (LCSC)-like cells. Figure S13. Pyrrolidinedithiocarbamate (PDTC) induced senescence in squamous cell carcinoma (SCC)- and adenocarcinoma (AC)-derived lung cancer stem cell-like cells. Figure S14. Inhibition of upstream pro-inflammatory signaling using lenalidomide only significantly decreased survival of squamous cell carcinoma (SCC)- not adenocarcinoma (AC)-derived lung cancer stem cell-like cells. Figure S15. Pyrrolidinedithiocarbamate (PDTC) treatment slightly decreased nuclear RelA of squamous cell carcinoma (SCC)- and adenocarcinoma (AC)-derived lung cancer stem cell-like cells. Table S1. Cell population-specific donor information. Table S2. Analysis of therapeutical relevant mutations.
