# Supplementary material for: Novel Primary Human Cancer Stem-Like Cell Populations from Non-Small Cell Lung Cancer: Inhibition of Cell Survival by Targeting NF-κB and MYC Signaling
Source: Cells. 2021 Apr 27;10(5):1024. doi: 10.3390/cells10051024 (PMC8145874; doi:10.3390/cells10051024)
Supplement: Supplementary file 1 [file cells-10-01024-s001.zip › cells-1130415-supplementary.pdf]

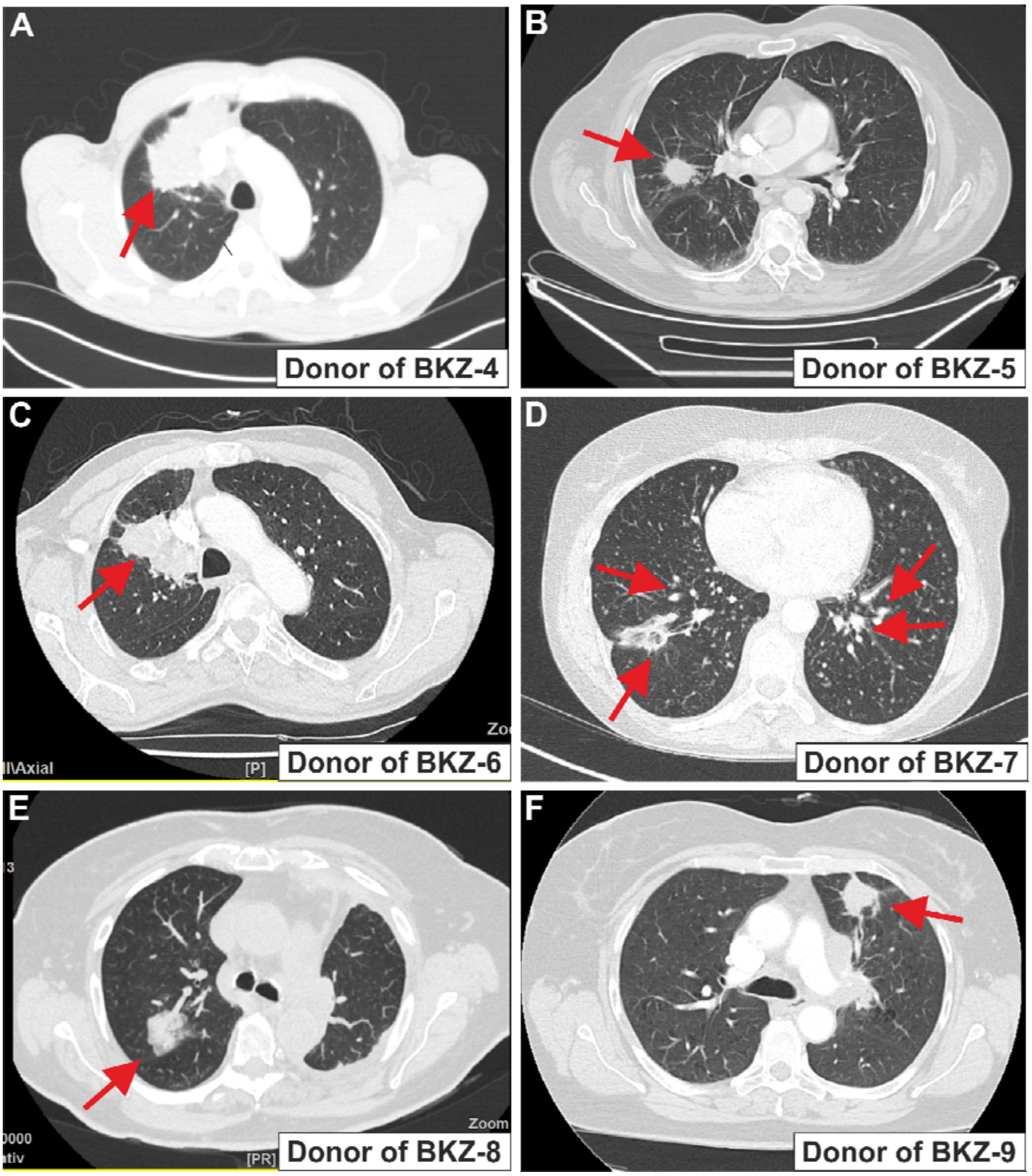

Figure S1. Clinical imaging of the different donors of the six isolated lung cancer stem cell-like cell populations. Computerized tomography (CT)-scan of the donors of **(A)** BKZ-4 and **(B)** BKZ-5 revealed a non-keratinizing squamous cell carcinoma of grade 2 centrally located in the right upper lobe of the lung, respectively (red arrows). **(C)** CT-scan of the donor of BKZ-6 showed a keratinizing squamous cell carcinoma of grade 2 within the upper right lobe of the lung (red arrow). **(D)** Clinical imaging of the donor of BKZ-7 revealed a highly metastatic invasive adenocarcinoma of the lung, with various pulmonary metastases (red arrows). **(E)** Clinical analysis of BKZ-8-donor showed a multifocal invasive adenocarcinoma of the left upper lobe of the lung of grade 2. **(F)** Clinical imaging of the donor of BKZ-9 depicted a grade 2 invasive adenocarcinoma of the upper left lobe of the lung.

Table S1. Cell population-specific donor information.

Donor of cell population	Tumor classification	Location of the Tumor / WHO grade	Sex	Age
BKZ-4	Non-keratinizing squamous cell carcinoma	right upper lobe / GII	Male	67
BKZ-5	Non-keratinizing squamous cell carcinoma	right upper lobe / GII	Male	79
BKZ-6	Keratinizing squamous cell carcinoma	right upper lobe / GII	Male	74
BKZ-7	Highly metastatic adenocarcinoma of the lung	Both sides of the lung	Female	50
BKZ-8	Multifocal adenocarcinoma	left upper lobe / GII	Female	61
BKZ-9	adenocarcinoma	left upper lobe / GII	Female	49

Table S2. Analysis of therapeutical relevant mutations.

Donor of cell population	*EGFR-* mut.	*KRAS-* mut.	*BRAF-* mut.	STK11 mut.	ALK	ROS1	RET	MET	NTRK1/ NTRK2/ NTRK3
BKZ-4	n/a[1]	n/a	n/a	n/a	n/a	n/a	n/a	n/a	n/a
BKZ-5	n/a	n/a	n/a	n/a	n/a	n/a	n/a	n/a	n/a
BKZ-6	n/a	n/a	n/a	n/a	n/a	n/a	n/a	n/a	n/a
BKZ-7	Mut.[2]	WT	WT	WT	neg.[3]	neg.	neg.	normal	neg.
BKZ-8	WT	WT	WT	WT	neg.	neg.	neg.	normal	neg.
BKZ-9	WT	Mut.	WT	Mut.	neg.	neg.	neg.	n/e[4]	neg. / n/e / n/e

[1] n/a = not announced; [2] mut. = mutation, WT = wildtype, [3] neg. = negative, [4] n/e = not evaluable.

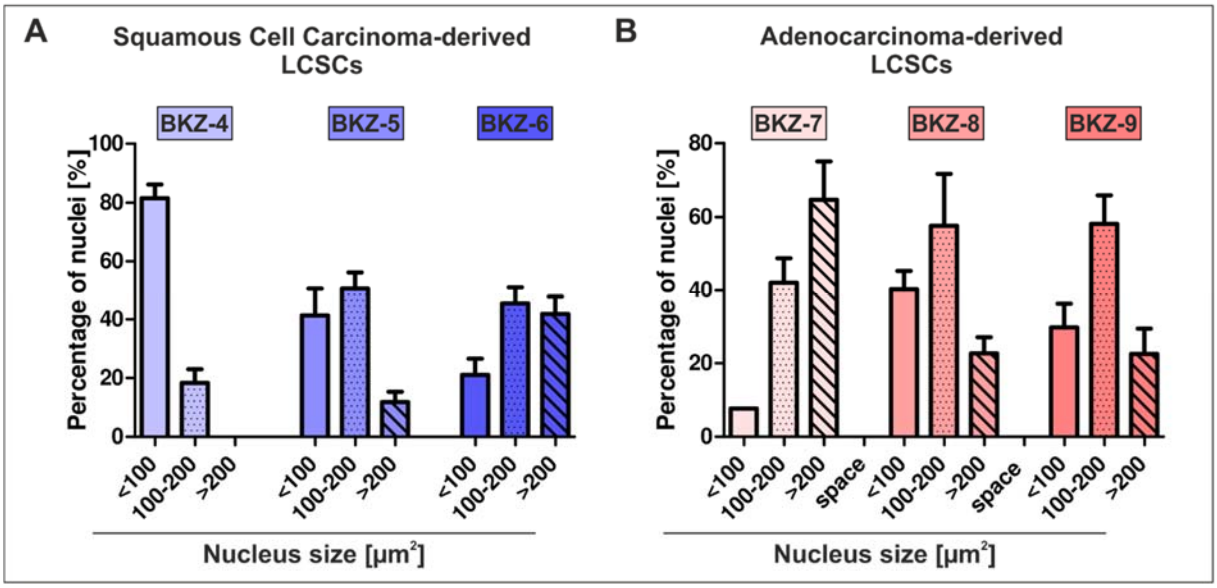

Figure S2. SCC- and AC-derived LCSC-like cells were heterogeneous in size. Quantification of the nucleus size of **(A)** SCC- and **(B)** AC-derived cells using at least five randomized immunocytochemical images revealed heterogeneity for all six cell populations.

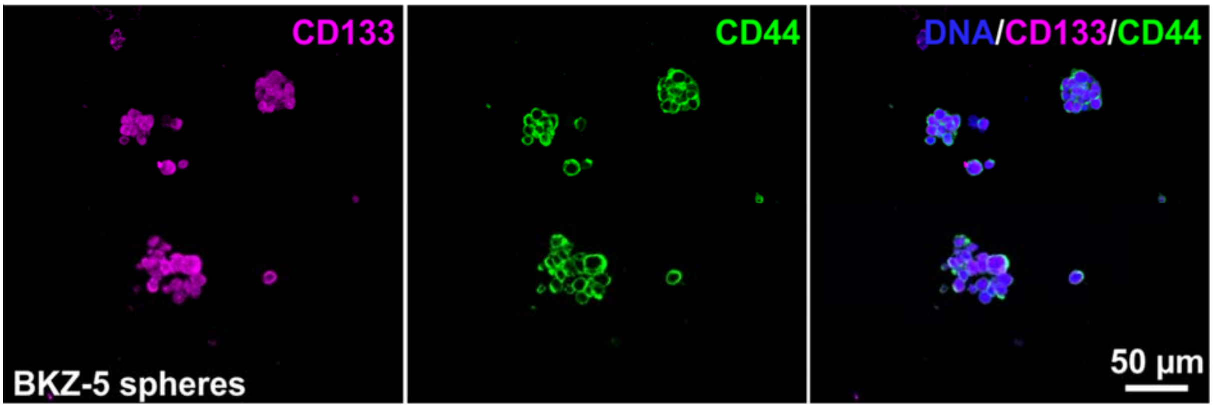

Figure S3. BKZ-5-derived spheres expressed cancer stem cell markers Prominin-1 (CD133) and CD44-antigen (CD44). Immunocytochemical staining of spheres derived from BKZ-5 revealed expression of cancer stem cell markers CD133 and CD44. Nuclear counterstaining was performed using 4′,6-diamidino-2-phenylindole.

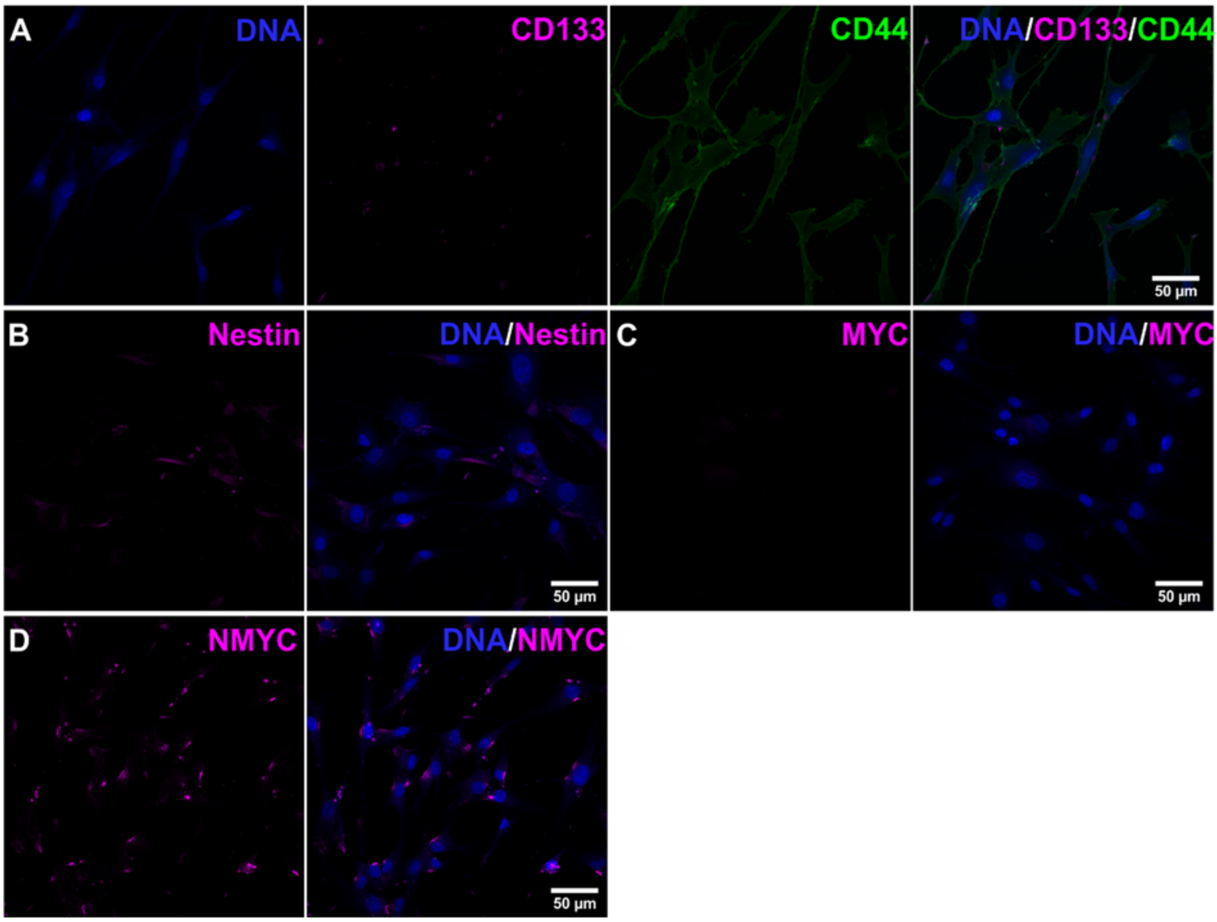

Figure S4. Immunocytochemical staining of cancer stem cell markers in adult human dermal fibroblasts (HDF). HDF served as biological negative control for the expression of cancer stem cell markers **(A)** Prominin-1(CD133)/CD44-antigen (CD44) **(B)** Nestin, **(C)** myc-proto-oncogene (MYC) and **(D)** N-myc proto-oncogene (NMYC). Nuclear counterstaining was performed using 4′,6-diamidino-2-phenylindole.

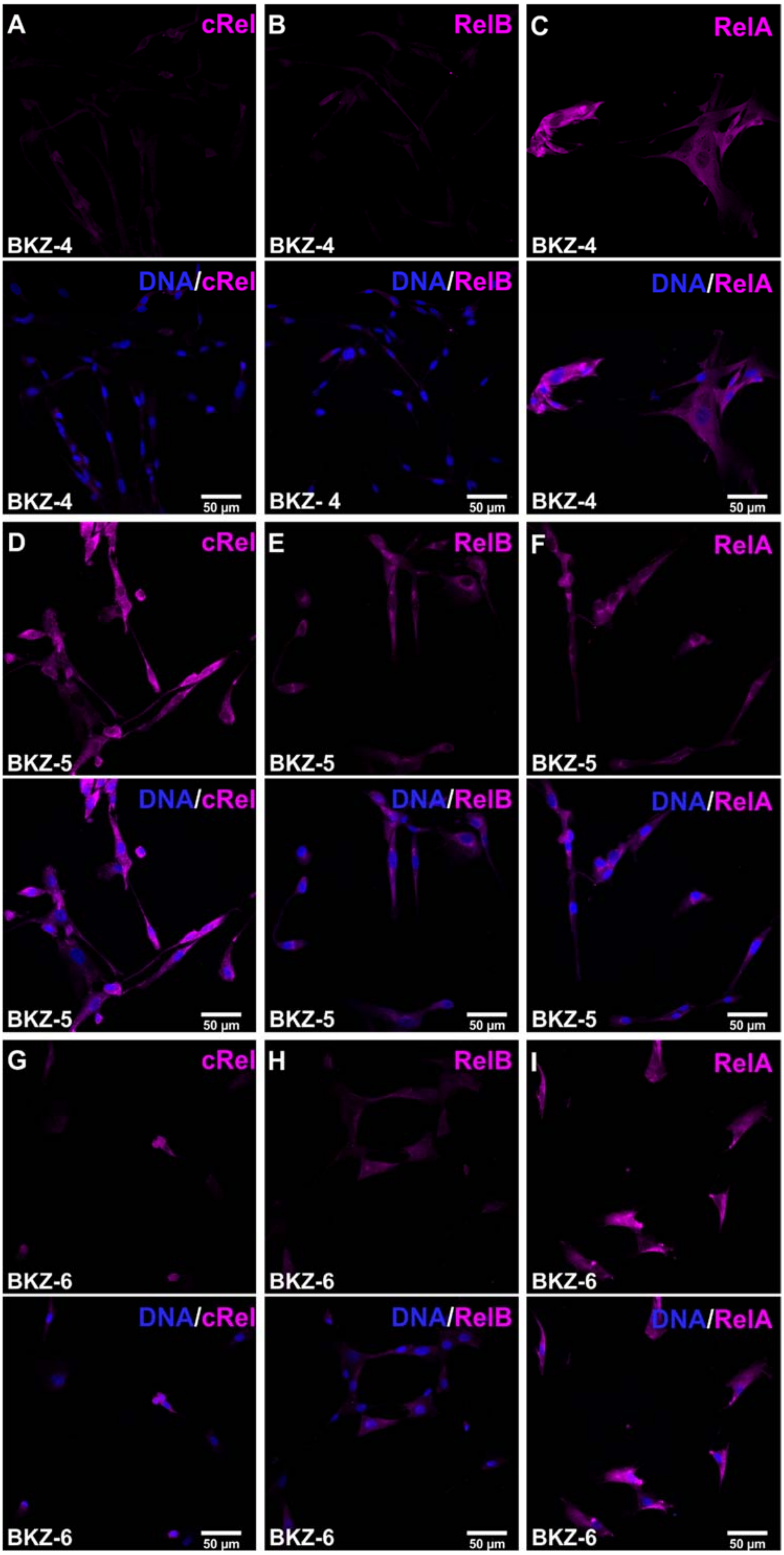

A
cRel
BKZ-4
DNA/cRel
BKZ-4
50 µm
B
RelB
BKZ-4
DNA/RelB
BKZ- 4
50 µm
C
RelA
BKZ-4
DNA/RelA
BKZ-4
50 µm
D
cRel
BKZ-5
DNA/cRel
BKZ-5
50 µm
E
RelB
BKZ-5
DNA/RelB
BKZ-5
50 µm
F
RelA
BKZ-5
DNA/RelA
BKZ-5
50 µm
G
cRel
BKZ-6
DNA/cRel
BKZ-6
50 µm
H
RelB
BKZ-6
DNA/RelB
BKZ-6
50 µm
I
RelA
BKZ-6
DNA/RelA
BKZ-6
50 µm

Figure S5. Squamous cell carcinomas-derived lung cancer stem cell-like cells BKZ-4, BKZ-5 and BKZ-6 all expressed NF-κB subunits RelA, RelB and cRel. Immunocytochemical analysis of the protein expression of the three NF-κB subunits cRel, RelB and RelA revealed an expression of RelA within all (**C**) BKZ-4, (**F**) BKZ-5 and (**I**) BKZ-6 cells. Protein expression of cRel and RelB seem to be more heterogeneous among the SCC-derived cell populations, with (**A**,**B**) BKZ-4 and (**G**,**H**) BKZ-6 revealing only a slight cRel and RelB protein expression and (**D**,**E**) BKZ-5 showing a relatively high expression of cRel and RelB. Protein expression of all subunits was predominantly cytosolic.

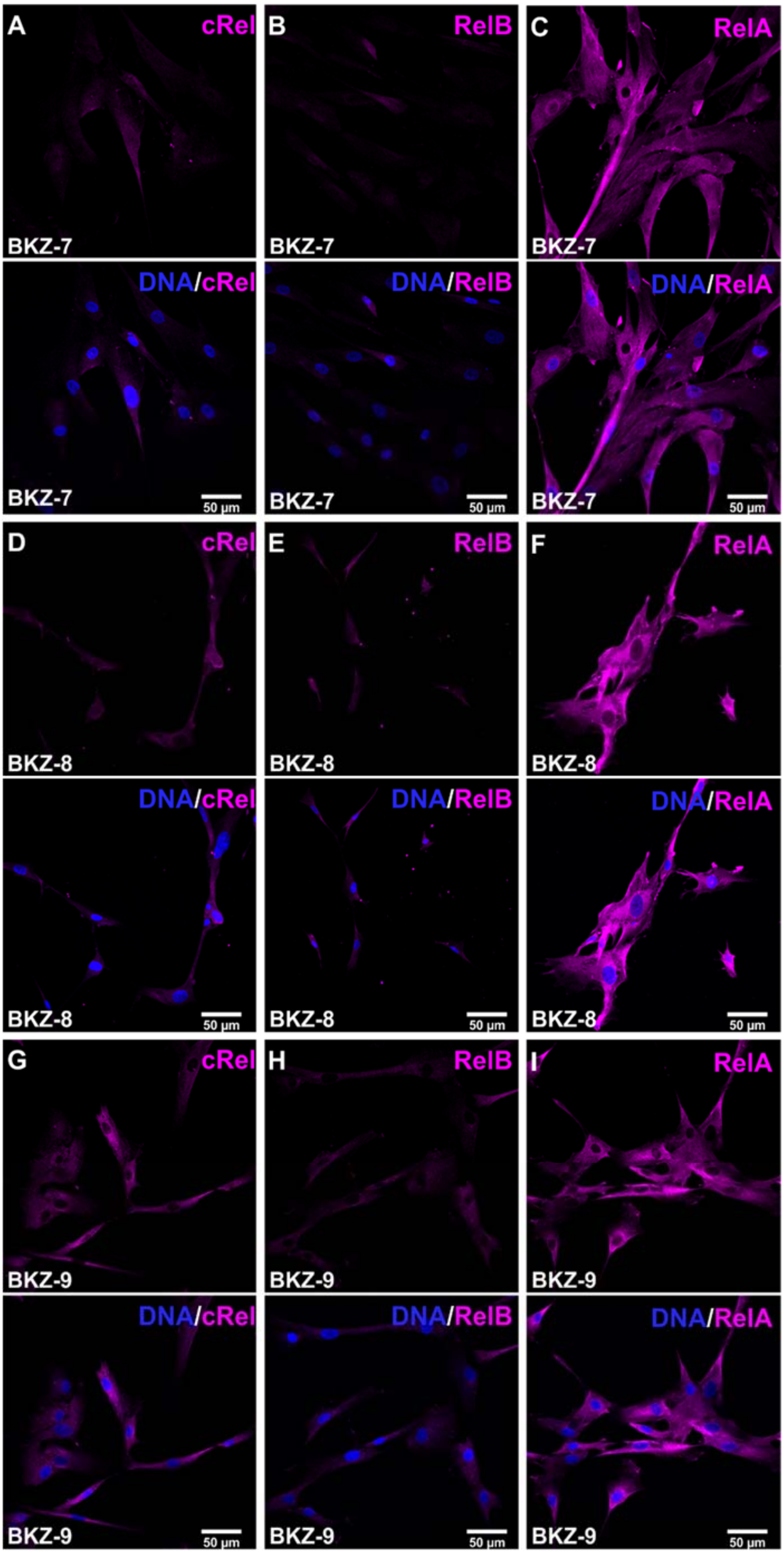

Figure S6. Adenocarcinoma-derived lung cancer stem cell-like cells all predominantly expressed NF-κB subunits RelA, RelB and cRel. Immunocytochemical analysis of the protein expression of NF-κB cRel, RelB and RelA showed a relatively high expression of RelA for **(C)** BKZ-7, **(F)** BKZ-8 and **(I)** BKZ-9.

Additionally, **(A,D,G)** cRel and **(B,E,H)** RelB were expressed as well, even though the expression was relatively low. Protein expression of all subunits was predominantly cytosolic.

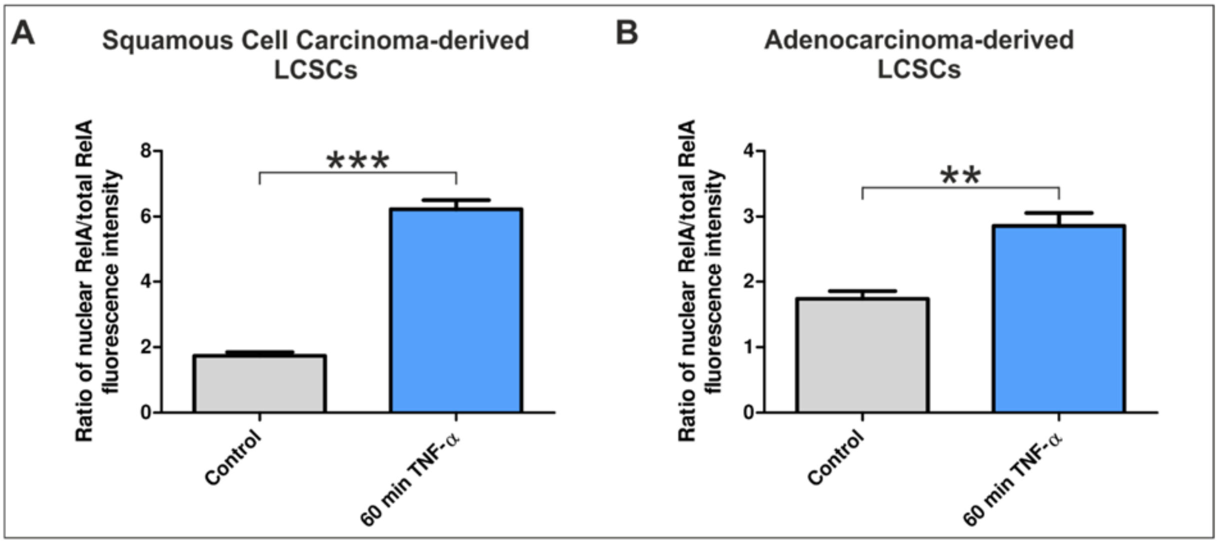

Figure S7. Stimulation with tumor necrosis factor α (TNF-α) shifted cytoplasmic NF-κB RelA into the nucleus of squamous cell carcinoma- and adenocarcinoma-derived lung cancer stem-like cells. Quantification of the ratio of fluorescence intensity of nuclear RelA to total RelA after 60 min TNF-α-treatment revealed a significant increase in ratio, representing a shift of basal cytoplasmic RelA into the nucleus of stimulated cells. Analysis of **(A)** SCC-derived cell populations as well as **(B)** AC-derived ones depicted a significant increase in ratio after 60 min exposure to TNF-α. Non-parametric Mann-Whitney-test ($p \leq 0.05$). n = 3, ** $p \leq 0.01$, *** $p \leq 0.001$. Mean ± SEM (standard error of the mean).

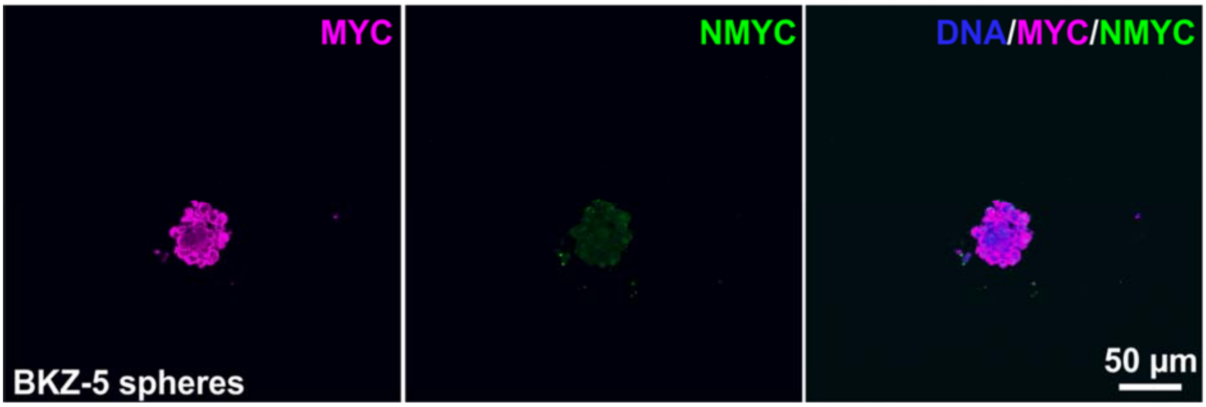

Figure S8. BKZ-5-derived spheres expressed myc proto-oncogene (MYC) and slightly N-myc proto oncogene (NMYC). Immunocytochemical staining of spheres derived from BKZ-5 revealed strong expression of MYC and slight expression of NMYC. Nuclear counterstaining was performed using 4’,6-diamidino-2-phenylindole.

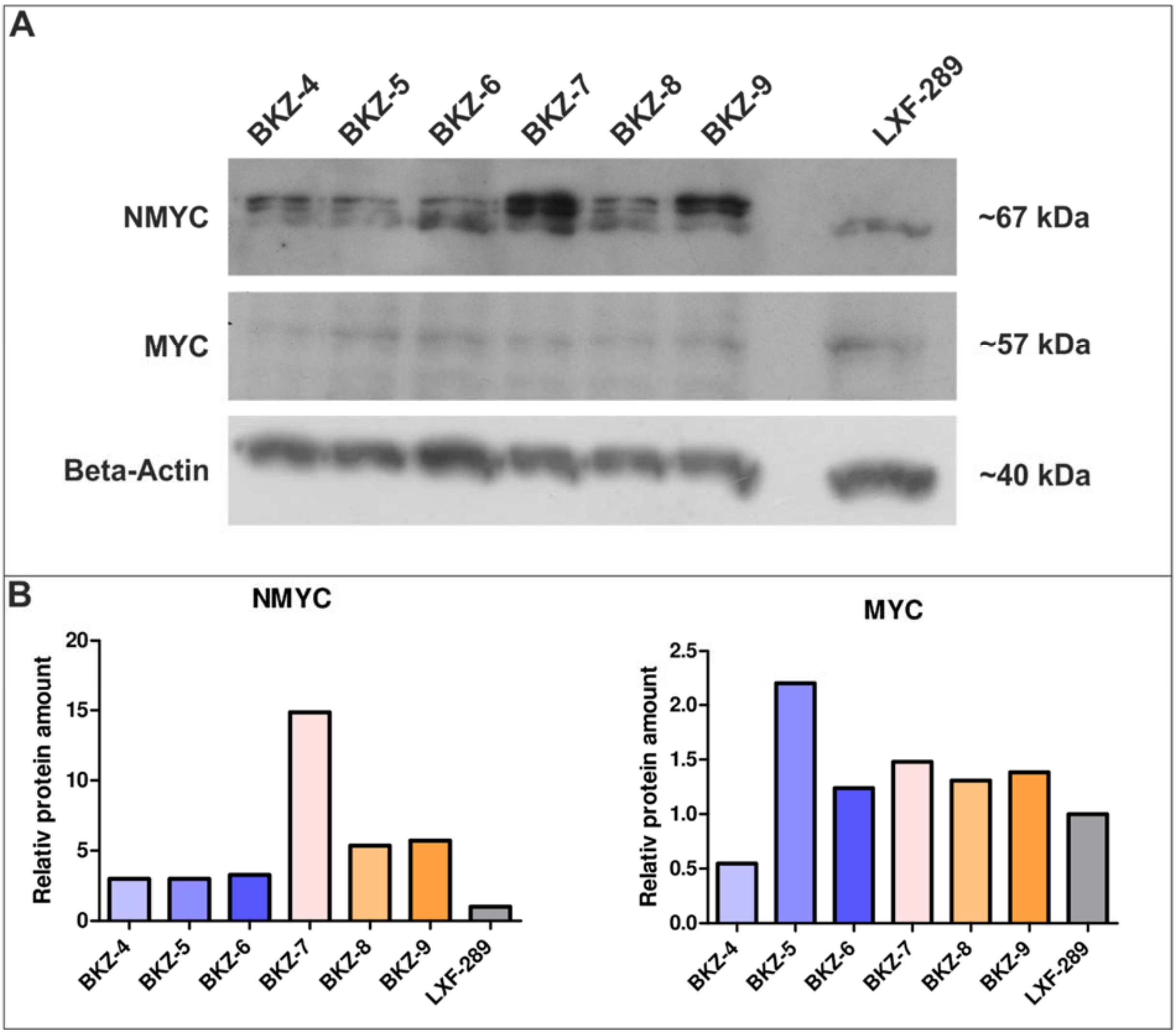

Figure S9: Western blot analysis of the protein levels of NMYC and MYC within BKZ populations and LXF-289 cells. **(A)** Immunoblots revealed expression of NMYC and MYC protein within all BKZ populations and LXF-289. **(B)** Quantification of MYC and NMYC protein showed higher expression in BKZ populations compared to LXF-289, except for BKZ-4 for MYC.

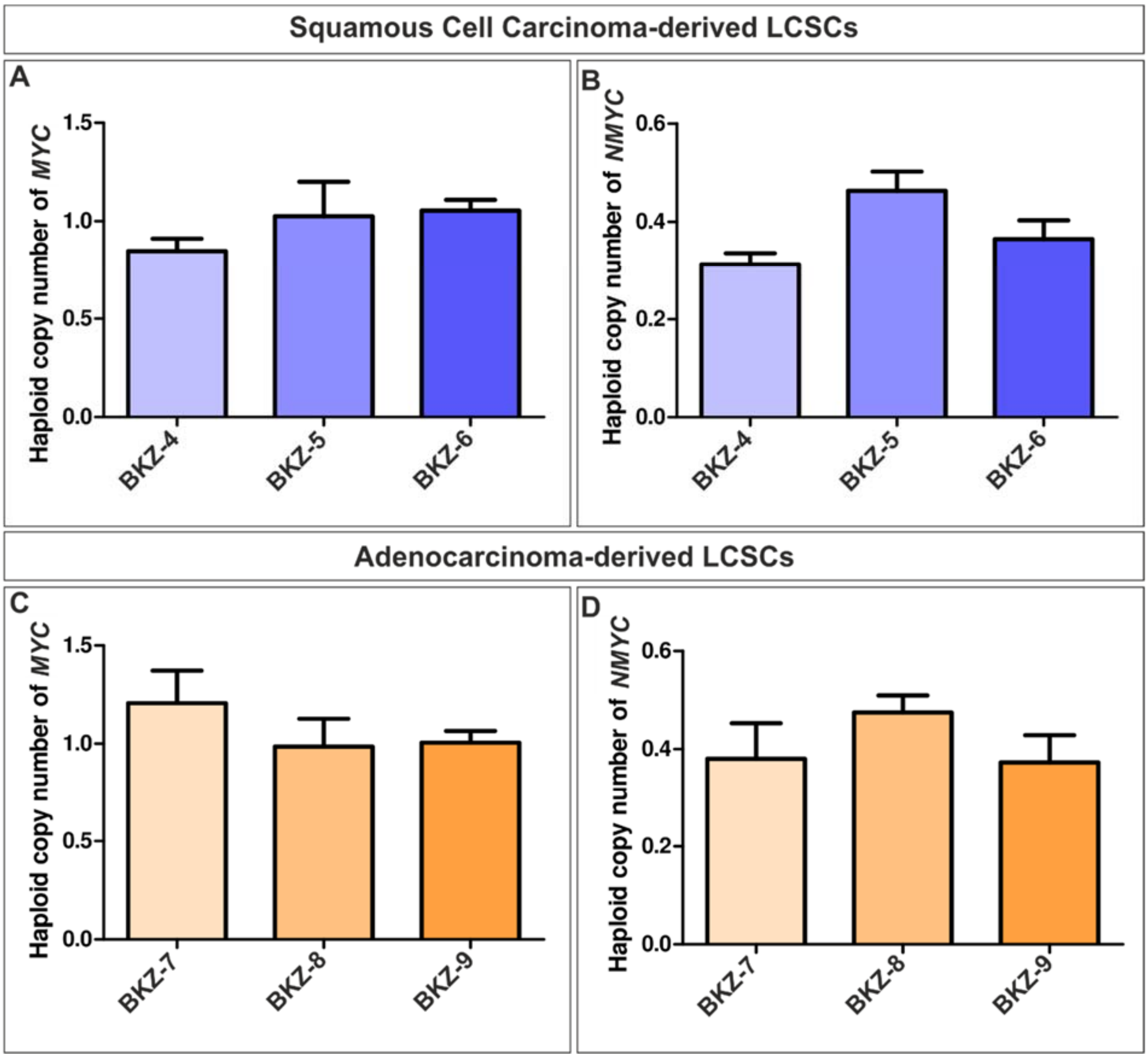

Figure S10: Squamous cell carcinoma (SCC)- and adenocarcinoma (AC)-derived LCSC-like cells all revealed a normal copy number for myc proto-oncogene (*MYC)* and N-myc proto-oncogene (*NMYC)*. Analysis of the haploid copy number of the two oncogenes showed normal copy number for **(A/C)** *MYC* and **(B/D)** *NMYC* for all three SCC-derived LCSCs as well as for AC-derived ones.

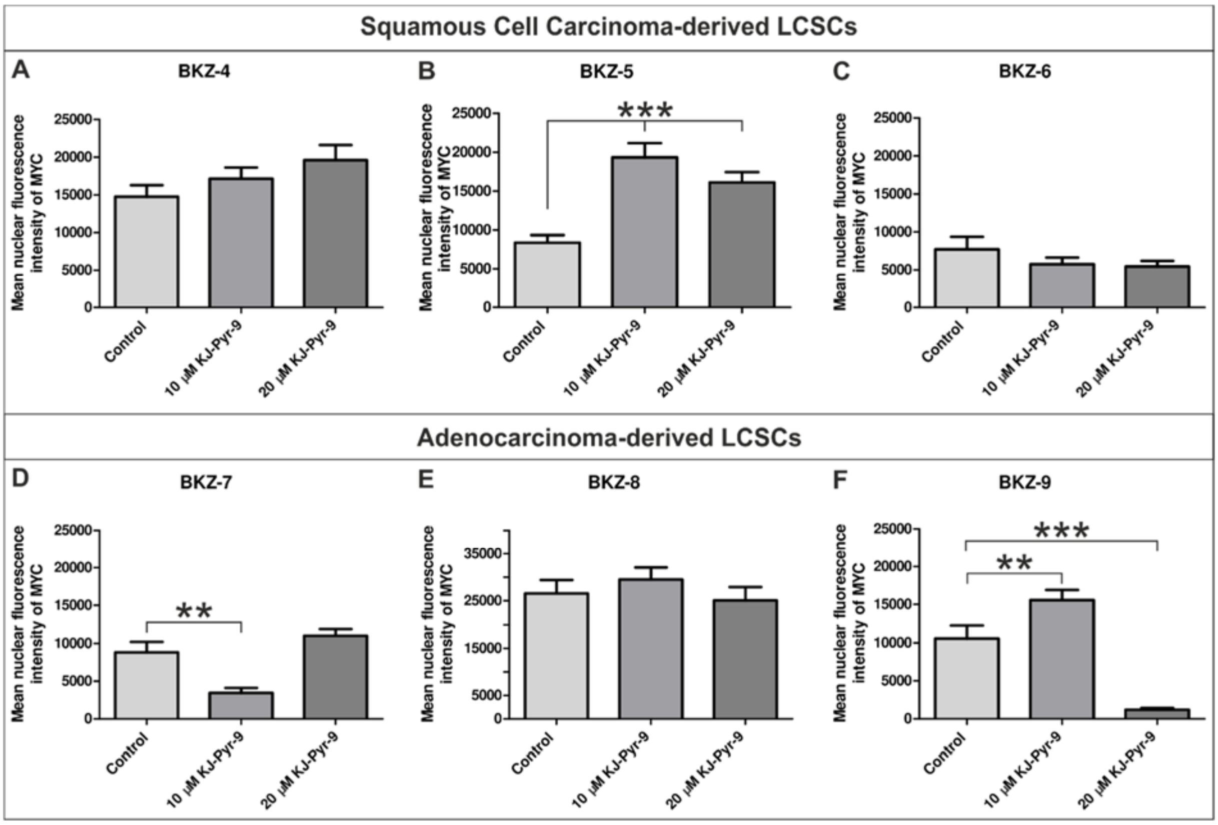

Figure S11. Treatment of non-small cell lung cancer-derived cell populations with KJ-Pyr-9 led to heterogeneous nuclear localization of myc proto-oncogene (MYC). Quantification of the nuclear fluorescence intensity of MYC after 24h-stimulation with 10 μM and 20 μM KJ-Pyr-9 depicted no clear trend concerning the nuclear translocation of MYC for **(A–C)** squamous cell carcinoma- as well as **(D–F)** adenocarcinoma-derived lung cancer stem cell-like cells. Non-parametric Mann-Whitney-test ($p \leq 0.05$). n = 3, ** $p \leq 0.01$, *** $p \leq 0.001$. Mean ± SEM (standard error of the mean).

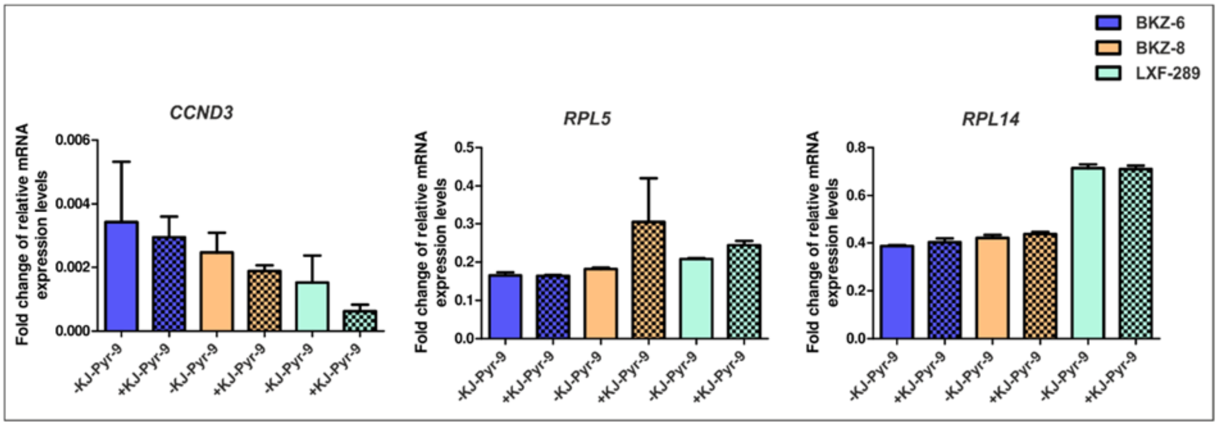

Figure S12: Analysis of putative MYC-target genes in lung cancer stem cell (LCSC)-like cells. Cyclin D3 (*CCND3*), ribosomal protein L5 (*RPL5*) and ribosomal protein L14 (*RPL14*) are not regulated with statistical significance by the application of 20 μM KJ-Pyr-9 in BKZ-5, BKZ-8 or LXF-289.

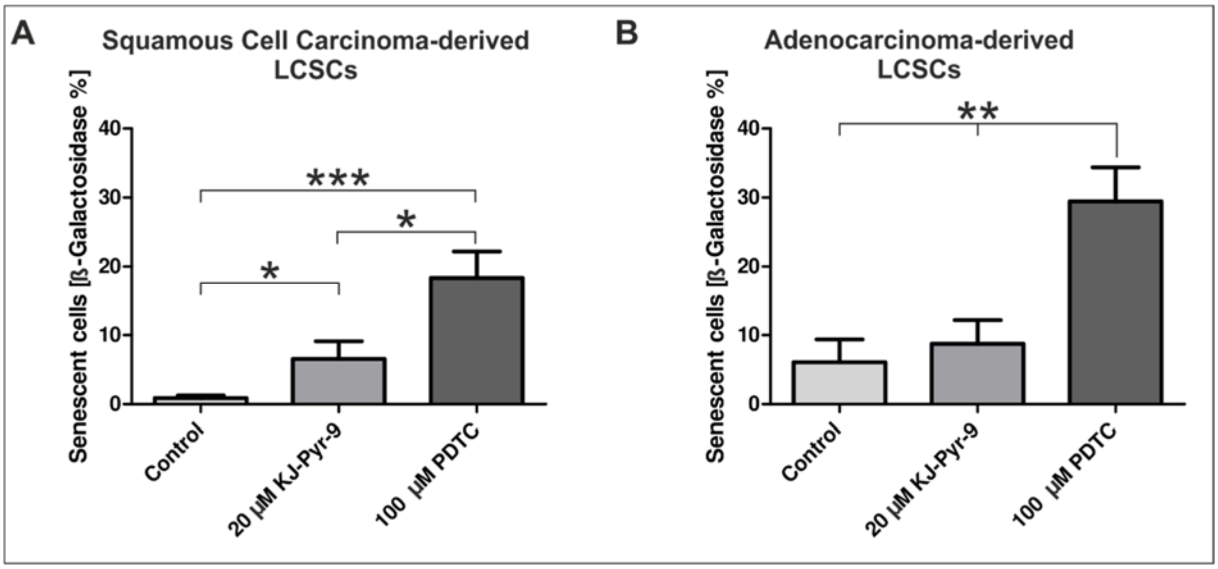

Figure S13. Pyrrolidinedithiocarbamate (PDTC) induced senescence in squamous cell carcinoma (SCC)- and adenocarcinoma (AC)-derived lung cancer stem cell-like cells. Quantification of senescence-associated β-galactosidase positive cells revealed a significant increase for senescent cells after PDTC-treatment for **(A)** SCC- and **(B)** AC-derived cell populations. Additionally, stimulation with 20 μM KJ-Pyr-9 led to a significant increase for SCC-derived senescence cells, but not for AC-derived cells. Unpaired t-test ($p \leq 0.05$). n = 3, * $p \leq 0.05$, ** $p \leq 0.01$, *** $p \leq 0.001$. Mean ± SEM (standard error of the mean).

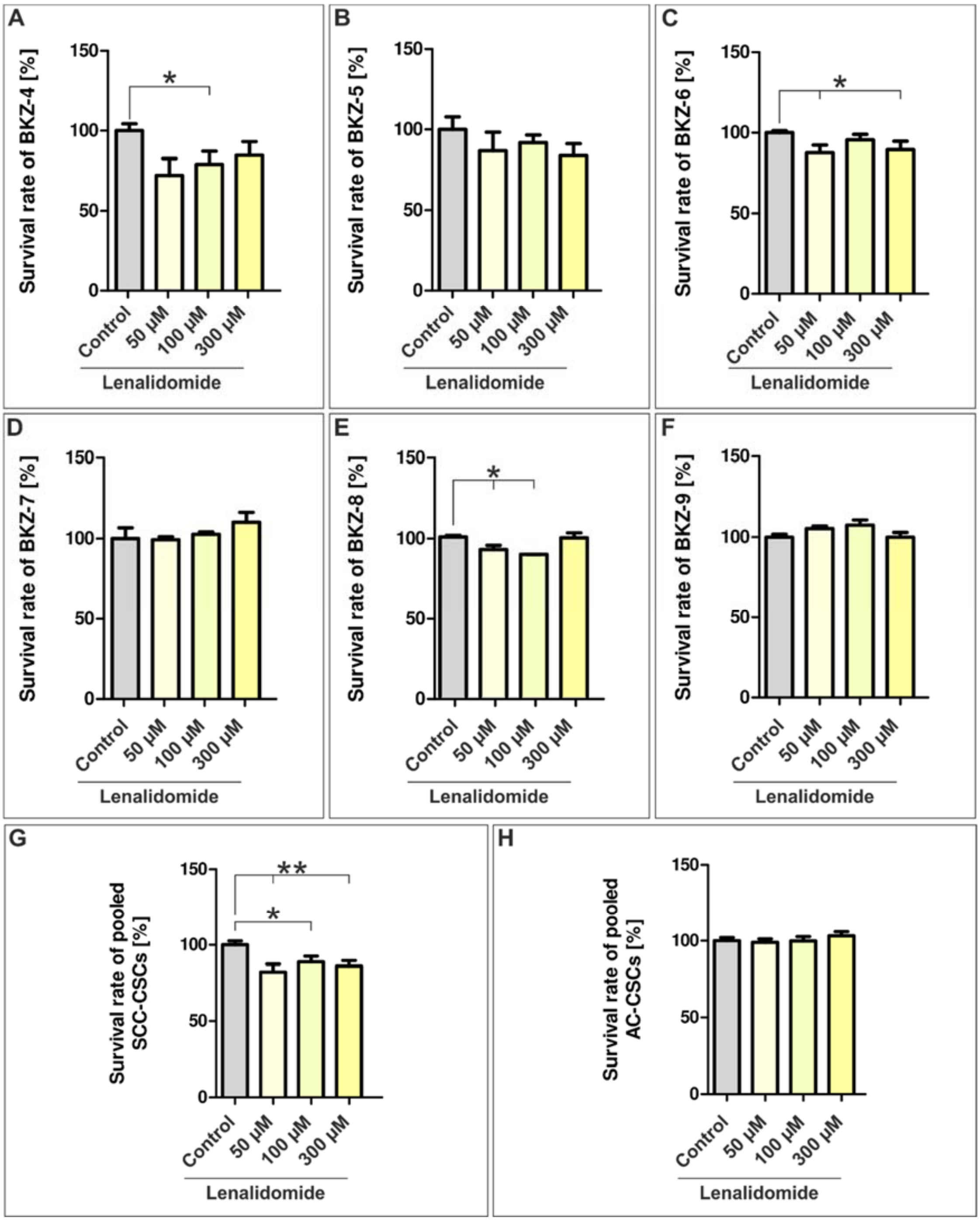

Figure S14. Inhibition of upstream pro-inflammatory signaling using lenalidomide only significantly decreased survival of squamous cell carcinoma (SCC)- not adenocarcinoma (AC)-derived lung cancer stem cell-like cells. To analyze the influence of the NF-κB inhibitor lenalidomide cells were treated with lenalidomide (50 µM, 100 µM, 300 µM) and cellular viability was measured using Orangu™. Quantification of normalized survival of **(A)** BKZ-4, **(B)** BKZ-5 and **(C)** BKZ-6 revealed a significantly survival-decreasing effect upon lenalidomide treatment for BKZ-4 and BKZ-6, with a survival-decreasing tendency for BKZ-5. Statistical analysis of the normalized survival rate of **(D)** BKZ-7, **(E)** BKZ-8 and **(F)** BKZ-9 after lenalidomide-exposure only revealed a significantly reduced survival for BKZ-8, with no effect on survival of BKZ-7 and BKZ-9. **(G)** Quantification of merged data of all three SCC-derived lung cancer stem cell-like cell populations revealed a survival decreasing effect, even though no dose dependent effect was detectable. **(H)** Analysis of merged data of all three AC-derived lung cancer stem cell-like cell populations showed no significant effect on cell survival upon lenalidomide-treatment. Non-parametric Mann-Whitney-test (**A–F**, $p \leq 0.05$). Unpaired t-test (**G,H**, $p \leq 0.05$). n = 3, * $p \leq 0.05$, ** $p \leq 0.01$. Mean ± SEM (standard error of the mean).

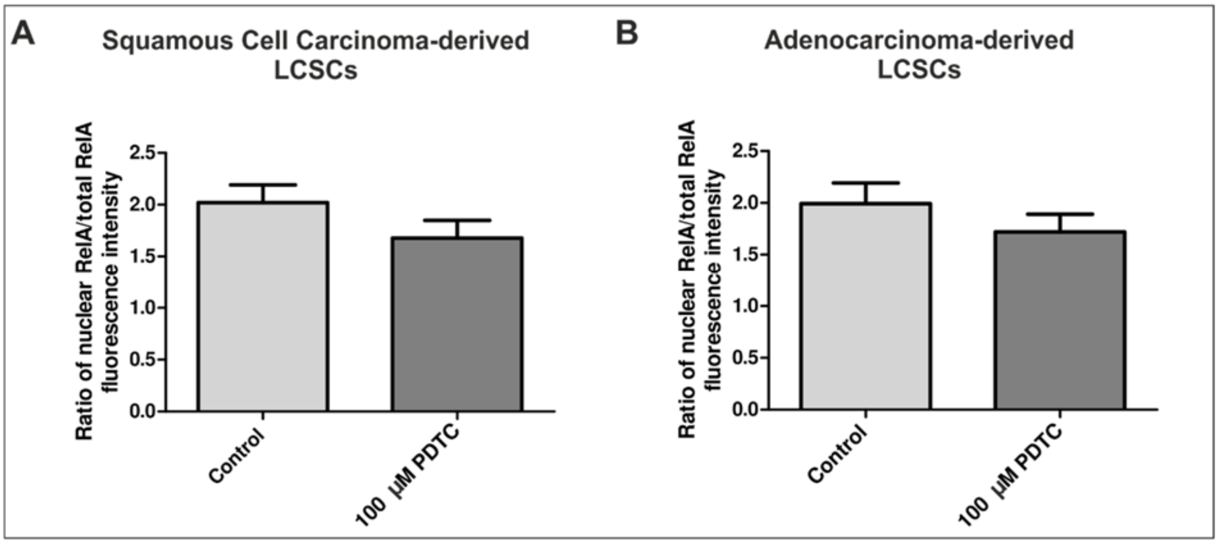

Figure S15. Pyrrolidinedithiocarbamate (PDTC)-treatment slightly decreased nuclear RelA of squamous cell carcinoma (SCC)- and adenocarcinoma (AC)-derived lung cancer stem cell-like cells. Quantification of the ratio of fluorescence intensity of nuclear RelA to total RelA depicted declining ratios upon PDTC-stimulation for **(A)** SCC- and **(B)** AC-derived cells, respectively. Certainly, the decrease in ratio was only a trend and not statistically significant. Non-parametric Mann-Whitney-test ($p \leq 0.05$). Mean ± SEM (standard error of the mean).
